# A Differentiation Transcription Factor Establishes Muscle-Specific Proteostasis in *Caenorhabditis elegans*

**DOI:** 10.1371/journal.pgen.1006531

**Published:** 2016-12-30

**Authors:** Yael Bar-Lavan, Netta Shemesh, Shiran Dror, Rivka Ofir, Esti Yeger-Lotem, Anat Ben-Zvi

**Affiliations:** 1 Department of Life Sciences and The National Institute for Biotechnology in the Negev, Ben-Gurion University of the Negev, Beer Sheva, Israel; 2 Regenerative Medicine and Stem Cell Research Center, Ben-Gurion University of the Negev, Beer Sheva, Israel; 3 Department of Clinical Biochemistry and Pharmacology and The National Institute for Biotechnology in the Negev, Ben-Gurion University of the Negev, Beer Sheva, Israel; The University of North Carolina at Chapel Hill, UNITED STATES

## Abstract

Safeguarding the proteome is central to the health of the cell. In multi-cellular organisms, the composition of the proteome, and by extension, protein-folding requirements, varies between cells. In agreement, chaperone network composition differs between tissues. Here, we ask how chaperone expression is regulated in a cell type-specific manner and whether cellular differentiation affects chaperone expression. Our bioinformatics analyses show that the myogenic transcription factor HLH-1 (MyoD) can bind to the promoters of chaperone genes expressed or required for the folding of muscle proteins. To test this experimentally, we employed HLH-1 myogenic potential to genetically modulate cellular differentiation of *Caenorhabditis elegans* embryonic cells by ectopically expressing HLH-1 in all cells of the embryo and monitoring chaperone expression. We found that HLH-1-dependent myogenic conversion specifically induced the expression of putative HLH-1-regulated chaperones in differentiating muscle cells. Moreover, disrupting the putative HLH-1-binding sites on ubiquitously expressed *daf-21(Hsp90)* and muscle-enriched *hsp-12*.*2(sHsp)* promoters abolished their myogenic-dependent expression. Disrupting HLH-1 function in muscle cells reduced the expression of putative HLH-1-regulated chaperones and compromised muscle proteostasis during and after embryogenesis. In turn, we found that modulating the expression of muscle chaperones disrupted the folding and assembly of muscle proteins and thus, myogenesis. Moreover, muscle-specific over-expression of the DNAJB6 homolog DNJ-24, a limb-girdle muscular dystrophy-associated chaperone, disrupted the muscle chaperone network and exposed synthetic motility defects. We propose that cellular differentiation could establish a proteostasis network dedicated to the folding and maintenance of the muscle proteome. Such cell-specific proteostasis networks can explain the selective vulnerability that many diseases of protein misfolding exhibit even when the misfolded protein is ubiquitously expressed.

## Author Summary

Molecular chaperones protect proteins from misfolding and aggregation. In multi-cellular organisms, the composition and expression levels of chaperones vary between tissues. However, little is known of how such differential expression is regulated. We hypothesized that the cellular differentiation that regulates the cell-type specific expression program may be involved in establishing a cell-type specific chaperone network. To test this possibility, we addressed the myogenic commitment transcription factor HLH-1 (CeMyoD) that converts embryonic cells to muscle cells in *Caenorhabditis elegans*. We demonstrated that HLH-1 regulates the expression of muscle chaperones during muscle differentiation. Moreover, we showed that HLH-1-dependent expression of chaperones is required for embryonic development and muscle function. We propose that cellular differentiation results in cell-specific differences in the chaperone network that may be detrimental in terms of the susceptibility of neurons and muscle cells to protein misfolding diseases.

## Introduction

Molecular chaperones are a diverse group of highly conserved proteins that evolved to cope with protein quality control challenges [1–3]. The cellular chaperone machinery is involved in a multitude of cellular functions, including *de novo* folding, assembly and disassembly of protein complexes, protein translocation across membranes, assisting proteolytic degradation and unfolding and reactivation of stress-denatured proteins [1, 3, 4]. The function and specificity of a chaperone-based reaction can be mediated by co-chaperones that choose the substrate, present it to the chaperone, and then coordinate cycles of binding and release by the chaperone in a manner that facilitates polypeptide unfolding [5–7]. Acute stress or chronic expression of metastable proteins leads to the accumulation of misfolded proteins that disrupts cellular protein homeostasis (proteostasis). Misfolded proteins continually occupy the chaperone machinery, such that overwhelming this machinery results in a shortage of chaperones for other cellular functions [8–12]. Activation of stress responses, such as the heat shock response, can induce chaperone genes, (chaperone and co-chaperone) expression and restore proteostasis [13]. However, this activation is also regulated by cell non-autonomous signals that can inhibit or induce a heat shock response regardless of protein damage [14]. Although chaperone over-expression often alleviates misfolded protein-associated toxicity [2, 15], accumulation of chaperones and activation of the heat shock response can also be detrimental to organismal health [12, 16–22], possibly by disrupting sub-networks of chaperones and co-chaperones [23–25].

The chaperone network in unicellular eukaryotes consists of two separately regulated chaperone sets, where one is co-regulated with the translational apparatus and one is stress-induced [26]. In multi-cellular eukaryotes, however, the complexity of the chaperone network is increased, with expression of components of the proteostasis network being highly heterogeneous between tissues, as well as dependent on age [2, 27]. Thus, the chaperone network may parallel the diverse composition of the proteome and its cellular folding requirements. However, it remains unknown how the expression of cell type-specific or ubiquitously expressed chaperones is regulated in different tissues. We reasoned that if chaperones expression is regulated in a cell-specific manner then differentiation transcription factors could play a role in defining the proteostatic network.

Muscle differentiation in *Caenorhabditis elegans* provides a well-studied case of highly regulated changes in cellular proteome composition within a specific time window [28–31], as well as information on molecular chaperones associated with muscle function [32]. *C*. *elegans* development is determined by the essentially invariant somatic cell lineage, so that the 81 embryonic muscle cells of the organism arise in a deterministic manner [33]. Muscle gene expression starts ~300 min after the first division. By ~350 min, dorsal and ventral muscle quadrants are formed, followed by the organization of muscle components into sarcomeres, and then by contraction of myofilaments at ~420–450 min [34]. Failure to properly fold and assemble the myofilaments disrupts myogenesis (arrest at two-fold phenotype) and can result in embryonic lethality [34]. *C*. *elegans* body-wall muscle differentiation is dependent on the core myogenic transcription factor modules HLH-1 (MyoD), UNC-120 and HND-1. Ectopic expression of each of these transcription factors can convert early blastomeres into muscle-like cells. However, in their absence only morphogenesis is disrupted and muscle differentiation can still occur [28, 35–37]. These transcription factors regulate the expression of many muscle proteins, such as myosin and actin [28, 30].

Many sarcomeric proteins require chaperones for their folding and assembly [32]. For instance, myosin folding and assembly requires the coordinated functions of the Hsp90 chaperone machinery (Hsp90 and its co-chaperones STI1-AHA1-P23) and the myosin-specific chaperone UNC-45 [25, 32, 38]. Moreover, there are examples of muscle-specific diseases that are associated with mutations in a ubiquitously expressed chaperone, such as DNAJB6 associated with the limb-girdle muscular dystropy [18, 39]. Here, we examined whether muscle chaperone expression is regulated by HLH-1 during *C*. *elegans* myogenesis. We found that the expression of chaperone genes with putative HLH-1-binding sites is induced by HLH-1-dependent myogenic conversion. We then demonstrated that disrupting the putative E-box motifs at the promoters of such chaperones inhibited HLH-1-dependent expression. Moreover, reduced HLH-1 expression resulted in a limited muscle proteostasis capacity during embryogenesis, larval development and adulthood. Finally, we showed that modulating the levels of muscle chaperones impacted the folding environment of muscle cells, disrupting muscle function and embryogenesis. We thus concluded that the myogenic transcription factor HLH-1 can regulate the expression of chaperones required for the folding and assembly of muscle proteins, establishing a cell-specific proteostasis network to fit cellular needs. We propose that cell-specific differences in the proteostatic network may contribute to tissue-specific vulnerability to protein misfolding diseases.

## Results

### Putative HLH-1 occupancy sites are associated with muscle chaperones

**Fig 1:**
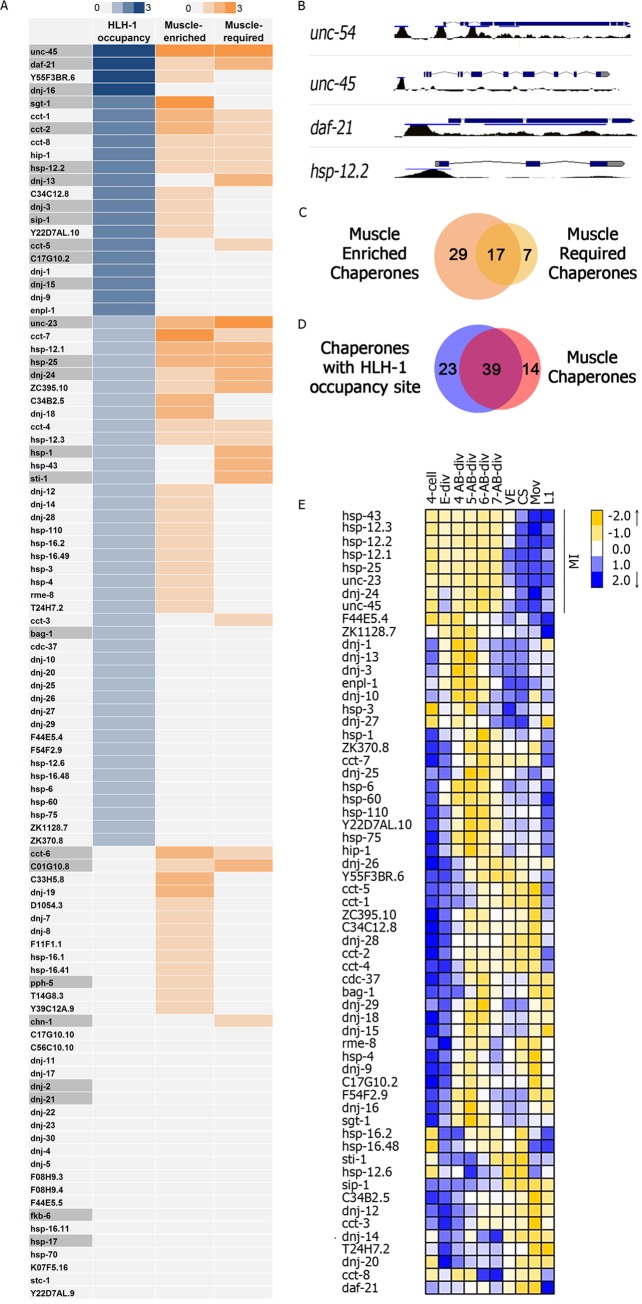
Promoter occupancy and transcriptional analysis of muscle chaperones reveals potential HLH-1-dependent regulation of chaperones. **(A)** A list of 97 *C*. *elegans* chaperones genes ranked according to potential for HLH-1 binding [29, 30] (HLH-1 occupancy), muscle-enrichment information [30, 31, 40] (Muscle-enriched) and literature-curated information [18, 25, 38, 39, 41–54] (Muscle-required) (see Methods). **(B)** HLH-1 occupancy sites associated with the promoter region of *unc-54(myosin heavy chain B)*, *unc-45*, *daf-21(Hsp90)* and *hsp-12*.*2(sHsp)* [29]. **(C)** Overlap between muscle-required and muscle-enriched chaperone sets. **(D)** Overlap between muscle-chaperones and chaperones with HLH-1 occupancy site sets. **(E)** Hierarchical clustering of the relative expression of 62 chaperone genes with HLH-1 occupancy sites across 10 developmental stages (at 4-cells, E cell division, 4^th^-7^th^ AB cell divisions, ventral enclosure (VE), comma stage (cs), first movement, and L1) [55]. MI marks the myogenesis-induced subset.

HLH-1 is the main myogenic transcription factor in *C*. *elegans*. To test whether chaperone expression is associated with cellular differentiation, we first assessed the potential of HLH-1 to regulate chaperone expression during muscle differentiation. Using chromatin immunoprecipitation and next-generation sequencing (ChIP-seq), two independent studies mapped the occupancy sites for this factor. One study used myogenic conversion, while the second used animals expressing HLH-1::GFP to increase HLH-1 detection [29, 30]. We used a set of 97 *C*. *elegans* chaperone genes [25] to ask whether there are putative HLH-1-binding sites associated with chaperone genes. Chaperone genes identified in at least one ChIP-seq experiment as being bound by HLH-1 were defined as chaperones with a HLH-1 occupancy site. This analysis resulted in a set of 62 chaperone genes (Fig 1A and S1 Table). The occupancy sites for these genes were found mainly in the promoter region, similar to other genes possessing HLH-1 occupancy sites [29, 30] (Fig 1B).

We ranked the 97 chaperone genes according to the number of independent ChIP-Seq experiments in which they were identified. Strong candidate genes, such as *unc-45* and *daf-21(Hsp90)*, were found to bind HLH-1 in all three ChIP-Seq experiments. Unlikely candidates included *hsp-17(sHsp)* and *fkb-6(FKBP)* for which an HLH-1-binding site was not identified (Fig 1A).

We then asked whether chaperones with HLH-1 occupancy sites are expressed in muscle cells. To define muscle-expressed chaperones, we considered three independent datasets of muscle-enriched genes: (1) An RNA-sequencing dataset of genes expressed in myogenic-converted embryos [30]; (2) a microarray dataset of genes expressed in muscle cells isolated by sorting cells from dissociated embryos expressing green fluorescence protein-tagged myosin (MYO-3::GFP) [31]; and (3) an mRNA dataset isolated from muscle cells at the first larval stage (L1) using mRNA-binding proteins expressed specifically in body-wall muscles [40]. This last dataset represents proteins that were expressed in functional muscle cells during post-embryonic development. Here, too, chaperones were ranked according to the number of datasets in which they were identified (Fig 1A). Combining these datasets, we identified 46 chaperones that were muscle-enriched (S1 Table).

Next, we used manual curation to identify muscle-required chaperones. The literature was scanned for reports of: (1) Chaperones shown *in vivo* to function in the folding of abundant muscle proteins, such as CCT/TRiC that is required for actin folding; (2) chaperones known to cause myopathies in humans, such as DNAJB6 (DNJ-24), as well as chaperones that affect *C*. *elegans* motility, such as UNC-23; and (3) chaperones that are localized to the sarcomere, such as HSP-12.1 [18, 25, 38, 39, 41–54] (S1 Table). This yielded 24 genes that were ranked according to the number of these criteria they matched (Fig 1A). Supporting a role for these chaperones in the folding and assembly of muscle proteins *in vivo*, the muscle-required set significantly overlapped with the muscle-enriched set (17 of 24, P = 0.008, Fisher exact test; Fig 1C). Most of the chaperone genes with HLH-1 occupancy sites were associated with muscle chaperones (enriched or required) (39 of 62, P = 0.025, Fisher exact test; Fig 1D), while chaperones with no identifiable HLH-1 occupancy site were not significantly associated with muscle chaperones (14 out of 35, P = 0.99, Fisher exact test). Thus, many muscle-enriched or -required chaperones have HLH-1 occupancy sites and can potentially be regulated by HLH-1.

Expression of well-established HLH-1-depndent muscle genes, such as myosins, is first observed ~300 min after the first division [34]. If HLH-1 occupancy sites are functional, chaperone genes that are bound by HLH-1 are expected to show a similar pattern of expression. While changes in muscle expression of ubiquitously expressed chaperones could be masked by their expression in other tissues [56], muscle specific or muscle-enriched chaperones are expected to show this pattern. We utilized the *C*. *elegans* developmental gene expression time course to characterize the myogenic-induced (MI) expression of genes during embryogenesis. This dataset, derived from whole embryos, records the expression of over 19,000 genes at ten different developmental stages over the course of embryogenesis [55]. Using this dataset, we first examined the expression dynamics of a set of known muscle genes that are also enriched in embryos showing increased muscle content upon myogenic conversion [30]. Of the 35 genes examined, the expression of 21 muscle-specific genes clustered into a single distinct developmental expression pattern (S1 Fig). The pattern showed little change in mRNA levels during early embryogenesis (<200 min) but a strong increase at the ventral enclosure stage (~290 min). We then asked whether the expression of some chaperone genes with HLH-1 occupancy site also follows this pattern of muscle protein expression. Of the 62 chaperone genes in this set, eight genes clustered into the MI expression pattern, of which seven were muscle-enriched and all eight were muscle-required (Fig 1A and 1E). As expected, ubiquitously expressed genes were not detected in this analysis. Thus, we were able to find a myogenic-induced expression pattern for a subset of HLH-1-associated chaperones also linked to muscle expression and function, supporting our hypothesis that muscle chaperones could be regulated by HLH-1 during muscle differentiation.

### Myogenic conversion modulates chaperone expression

**Fig 2:**
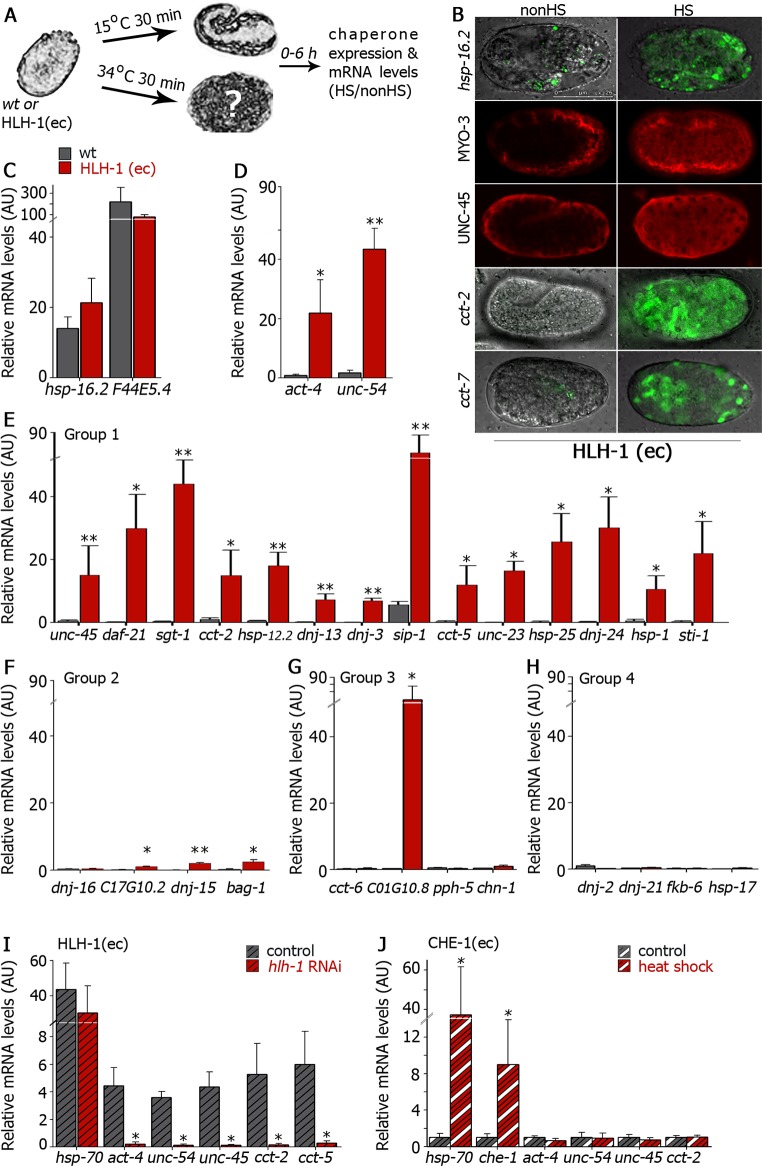
Myogenic conversion induced the expression of muscle chaperones. **(A)** Schematic representation of the experimental setup. Wild type (wt) or HLH-1(ec) embryos were untreated or subjected to heat shock (34°C, 30 min) and chaperone expression was examined. **(B)** Representative images (>90%) of the expression pattern of the indicated chaperones in untreated or heat shock embryos expressing HLH-1(ec) after a 6 h recovery. Scale bar is 25 μm. **(C-H)** Relative chaperone mRNA levels of heat shock-treated wild type (gray) or HLH-1(ec) (red) embryos (normalized to *T07A9*.*15*). Data are normalized to values obtained with untreated embryos and are presented as means ± SEM of at least 5 independent experiments. Gene groups were defined in S1 Table. **(I)** Relative mRNA levels of heat shocked HLH-1(ec) embryos grown on control (gray stripes) or *hlh-1* (red stripes) RNAi (normalized to *T07A9*.*15*). Data are relative to values obtained with untreated embryos and are presented as means ± SEM of at least 3 independent experiments. **(J)** Relative mRNA levels of untreated (gray stripes) or heat shocked (red stripes) CHE-1(ec) embryos (normalized to *T07A9*.*15*). Data are relative to values obtained with wild type embryos and are presented as means ± SEM of at least 3 independent experiments.

To experimentally test whether HLH-1 regulates chaperone expression during muscle differentiation, we first examined whether the ectopic expression of HLH-1 that induced myogenic conversion could also induce the expression of muscle chaperones in non-muscle cells. As such, we utilized animals expressing HLH-1 under the control of the inducible *hsp-16*.*41(sHsp)* promoter, HLH-1(ec) [28]. When such animals were exposed to a short heat shock (30 min at 34°C) during early embryogenesis, HLH-1 was ectopically expressed in all embryonic cells. Because heat shock induced the expression of heat shock genes, some of which are chaperones, we examined the expression of each gene in both HLH-1(ec) and wild type embryos with or without exposure to heat shock (Fig 2A). To control for heat shock-induced activation, animals expressing GFP under the control of the inducible chaperone promoter *hsp-16*.*2(sHsp)* were crossed with HLH-1(ec) animals and GFP expression was monitored. Upon heat shock, robust GFP expression was detected in most cells of the HLH-1(ec) embryos (Fig 2B), similar to wild type animals (S2A Fig). Likewise, heat shock genes, such as *hsp-16*.*2(sHsp)* and *F44E5*.*4(Hsp70)*, were similarly induced in both HLH-1(ec) and wild type embryos (Fig 2C). When we examined the expression of known HLH-1-regulated genes, such as myosin, by immuno-staining, heat shock-treated HLH-1(ec) embryos showed ectopic expression of myosin heavy chain A (MYO-3) in most cells of the embryo (Fig 2B) but not in wild type embryos (S2A Fig). In agreement, levels of actin *(act-4)* and myosin heavy chain B (*unc-54)* were induced in heat shock-treated HLH-1(ec) but not in wild type embryos (Fig 2D).

We then asked whether ectopic expression of HLH-1 and altered cellular fate affected the pattern and levels of expression of chaperone genes. We divided the chaperone list into four groups: (1) Chaperones with HLH-1 occupancy site identified in at least one experiment and that are muscle-associated (39 genes), or (2) that are not associated with muscle (21 genes); (3) chaperones with no identified HLH-1 occupancy site that are associated with muscle (14 genes), or (4) that are not associated with muscle (22 genes) (S1 Table). We then tested candidate genes (Fig 1A, gray shaded) from each group for myogenesis-dependent changes in expression induced by ectopic induction of HLH-1 (Fig 2E–2H). As expected, the expression of UNC-45, considered a HLH-1-specific substrate [30], was ectopically induced in most of the cells of the heat shocked HLH-1(ec) embryos (Fig 2B) but not of wild type animals (S2A Fig). To test for changes in expression of ubiquitously expressed chaperones, animals expressing GFP under the control of the *cct-2(Hsp60)* or *cct-7(Hsp60)* promoter were crossed with HLH-1(ec) animals and GFP fluorescence was monitored. Similar to the HLH-1 muscle genes tested, *cct-2(Hsp60)-* and *cct-7(Hsp60)*-dependent GFP expression was detected in most cells of the HLH-1(ec) embryos upon heat shock (Fig 2B) but not in wild type embryos (S2A Fig). Thus, myogenic-converted cells, differentiating into muscle cells, began to express chaperone genes. Indeed, mRNA of 14 muscle-associated chaperone genes with an HLH-1 occupancy site (group 1) were all induced (10–80 folds) in HLH-1(ec) embryos upon heat shock. This group included all MI chaperones tested (5 out of 8, Fig 1E), as well as ubiquitously expressed chaperones. In wild type embryos, in contrast, these chaperones expression levels (apart from *sip-1(sHsp)*) did not increase and indeed, some decreased following heat shock (Fig 2E and S2B Fig). Although *sip-1(sHsp)* levels increased in wild type embryos, its induction in HLH-1(ec) embryos was 10-fold higher (Fig 2E and S2B Fig). Chaperone genes with HLH-1 occupancy sites that were not associated with muscle (group 2) also showed increased levels in HLH-1(ec) embryos upon heat shock (3 of the 4 genes tested), albeit to a modest extent (1.5–3.5 fold). Thus, of the 18 chaperone genes with an identified HLH-1 occupancy site, 17 were significantly induced by ectopic expression of HLH-1 (Fig 2E and 2F and S2B and S2C Fig). In contrast, when we examined chaperones for which HLH-1 occupancy sites was not identified, regardless of their muscle association (groups 3 and 4), only one gene, *C01G10*.*8(Aha1)*, showed increased expression in HLH-1(ec) embryos upon heat shock (Fig 2G and 2H and S2D and S2E Fig). Thus, under conditions of induced myogenic conversion, when HLH-1-dependent muscle differentiation is activated, chaperones genes that were shown to bind HLH-1 are induced. This indicates that the majority of HLH-1 occupancy sites identified for chaperone genes are functional (24 out of 26 genes tested, i.e. 92%) and, similar to other muscle genes, are up-regulated when cells differentiate into muscle cells.

To verify that chaperone expression was due to HLH-1, HLH-1(ec) embryos from animals treated with control or *hlh-1* RNAi were heat shocked and changes in mRNA levels following heat shock were assessed. While expression of the inducible heat shock gene *hsp-70(Hsp70)* was unaffected by *hlh-1(RNAi)*, the induced expression of the muscle genes *act-4* and *unc-54* and the muscle chaperone genes *unc-45*, *cct-2(Hsp60)* and *cct-5(Hsp60)* was strongly reduced in *hlh-1(RNAi)*-treated HLH-1(ec) embryos, as compared to control RNAi-treated embryos (Fig 2I). Likewise, the expression of muscle and chaperone genes was not significantly induced when the transcription factor CHE-1 was ectopically expressed upon heat shock in embryos expressing *hsp-16*.*2*::*che-1*, although expression of *hsp-70(Hsp70)* and *che-1* was induced (Fig 2J). Thus, ectopic expression of HLH-1 that led to myogenic conversion, resulted in HLH-1-dependent induced expression of muscle chaperones in differentiating muscle cells.

### Mutations in putative HLH-1-binding motifs disrupt chaperone expression

A previous attempt to validate HLH-1 function using a HLH-1-binding site upstream of a minimal promoter was very limited in its ability to induce muscle expression, even of known muscle genes [29]. We, therefore, took a different approach to examine whether HLH-1 is required for chaperone expression during muscle differentiation. Accordingly, we asked how disruption of the HLH-1 E-box-binding motif at chaperone promoters would affect their expression in myogenic-converted embryos. Because the muscle-specific chaperone UNC-45 is considered one of the “gold standard” muscle genes regulated by HLH-1 [30], we examined two ubiquitously expressed chaperones. Specifically, DAF-21(Hsp90), a well-established myosin chaperone and HSP-12.2, a small HSP (sHsp) that showed a myogenic expression pattern during embryogenesis (Fig 1E). The DAF-21(Hsp90) HLH-1 occupancy site was identified in three independent ChIP-seq experiments and its binding peak at the promoter showed a clear E-Box consensus motif. The HSP-12.2(sHsp) HLH-1 occupancy site was identified in two independent ChIP-seq experiments and its binding peak at the promoter has two E-Box consensus motifs (S1 Table) [29, 30].

**Fig 3:**
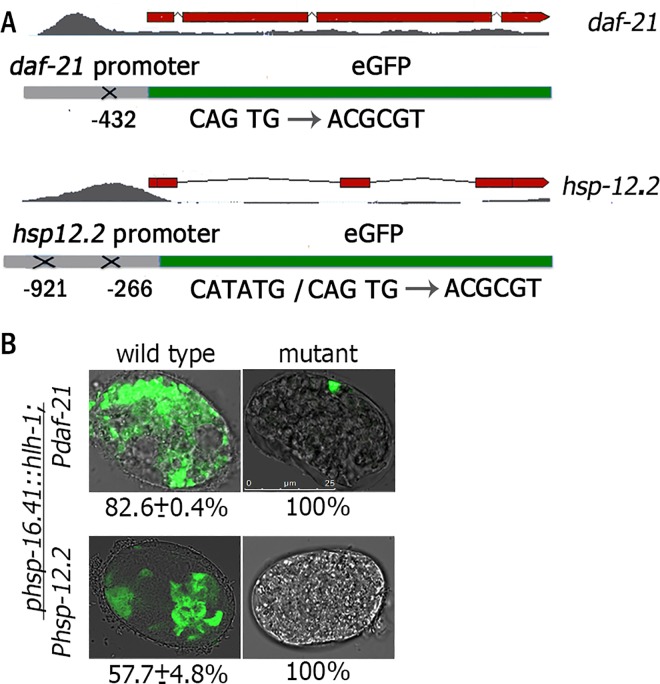
Mutation in the putative HLH-1-binding motifs of *daf-21(Hsp90)* and *hsp-12*.*2(sHsp)* promoters abolished their HLH-1-dependent expression. **(A)** Wild type or mutated promoter reporter constructs for *daf-21(Hsp90)*- or *hsp-12*.*2(sHsp)*-regulated GFP expression. (**B**) Representative images of HLH-1(ec) embryos expressing GFP under the regulation of the wild type or mutant *daf-21(hsp90)* (top) or *hsp-12*.*2(sHsp)* (bottom) promoter following heat shock (34°C, 30 min). Scale bar is 25 μm.

We constructed a transcription reporter containing the promoter region of *daf-21(Hsp90)* or *hsp-12*.*2(sHsp)* upstream of GFP (*daf-21*::*gfp* or *hsp12*.*2*::*gfp*) and mutated the E-box sequences (Fig 3A). These constructs were injected into HLH-1(ec) animals and stable transgenic animals were established. The expression of GFP in myogenic-converted embryos was then monitored following heat shock. In 82.6±0.4% of the *daf-21(Hsp90)* and 57.7±4.8% of the *hsp12*.*2(sHsp)* embryos carrying the wild type transcription reporters, GFP was ectopically expressed in most cells of the embryos upon heat shock. In contrast, GFP expression was undetected (less than 5 cells) in all the heat shock embryos carrying the mutated transcription reporters (P<0.05, Fig 3B). When *daf-21*::*GFP* embryos were allowed to develop, expression of both wild type and mutated constructs was observed in various tissues of the adult animals, including intestine and neurons (S3 Fig). However, we could not detect GFP expression in muscle cells of adult animals carrying the mutated transcription reporters. For example, no muscle expression was detected in animals carrying the mutated *daf-21*::*GFP* transcription reporter (n = 120), although wild type *daf-21*::*GFP* was expressed in muscle cells (S3 Fig). Thus, disrupting putative E-box sequences abolished the HLH-1-dependent regulation of *daf-21*::*GFP* and *hsp-12*.*2*::*GFP* in embryonic muscle cells, suggesting that HLH-1 occupancy sites at these promoters are transcriptionally functional and can drive muscle expression.

### Reduced HLH-1 levels limit muscle proteostasis capacity

**Fig 4:**
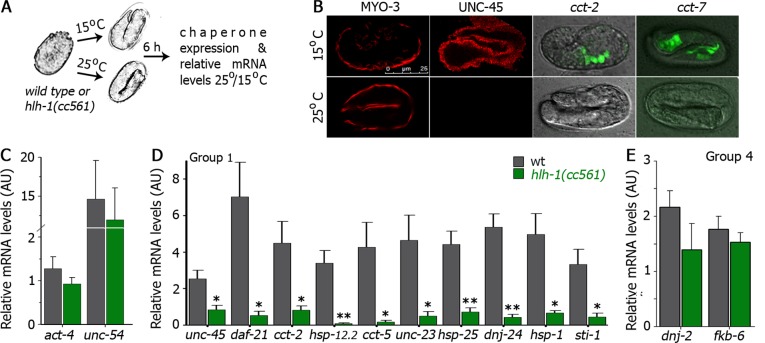
Reduced HLH-1 levels result in a decline in chaperone expression. **(A)** Schematic representation of the experimental setup. Wild type or *hlh-1(cc561)* embryos were grown at 15 or 25°C for 6 h and chaperone expression was examined. **(B)** Representative images (>90%) of the expression pattern of the indicated chaperones in *hlh-1(cc561)* embryos grown at 15 or 25°C. Scale bar is 25 μm. **(C-E)** Relative mRNA levels (25/15°C) of wild type (gray) or *hlh-1(cc561)* (green) embryos (normalized to *T07A9*.*15*). Data are presented as means ± SEM of 5 independent experiments.

To complement the approach taken above and to determine the contribution of HLH-1 to muscle proteostasis, we examined the effects of down-regulating *hlh-1* on chaperone expression during embryogenesis, using a truncation allele, *hlh-1(cc561)*. This nonsense (Glu222Stop) mutation does not affect HLH-1 function but results in temperature-dependent *hlh-1* mRNA clearance by the nonsense mRNA decay pathway and, therefore, temperature-dependent knockdown of HLH-1 levels [28, 57]. We thus asked whether the expression of chaperone genes with HLH-1 occupancy sites was affected in *hlh-1(cc561)* animals grown at 25°C, as compared to animals grown at 15°C. Wild type or *hlh-1(cc561)* embryos laid at 25°C were allowed to develop for 6 h. Protein expression and mRNA levels of muscle genes were then compared with those values obtained in embryos maintained at 15°C (Fig 4A). Some muscle proteins, including the major myosins and actins, were unaffected by *hlh-1(cc561)* because UNC-120 serves a partially overlapping function and can compensate for a loss of HLH-1 [30]. In agreement, immuno-staining of *hlh-1(cc561)* embryos with anti-MYO-3 antibodies showed a typical organization pattern in body-wall muscle cells in embryos grown at 15°C and 25°C (Figs 4B and S4A). The relative mRNA levels (25°C/15°C) of *act-4* and *unc-54* were also similar in *hlh-1(cc561)* and wild type animals (Fig 4C). In contrast, the localization of myosin chaperone UNC-45 was lost in *hlh-1(cc561)* embryos grown at 25°C and relative *unc-45* mRNA levels were reduced in *hlh-1(cc561)*, as compared to wild type embryos (Fig 4B and 4C and S4A Fig). Likewise, the expression of GFP under the control of the *cct-2(Hsp60)* or *cct-7(Hsp60)* promoter in *hlh-1(cc561)* embryos grown at 25°C was lost and the relative mRNA levels of different muscle chaperones shown to be regulated by HLH-1(ec) (group 1 and 2) were significantly reduced in *hlh-1(cc561)* embryos, as compared to wild type embryos (Fig 4B and 4D, S4A and S4B Fig). While the expression of *C01G10*.*8(Aha1)* that was induced in heat-shocked and treated HLH-1(ec) embryos was significant reduced (S4C Fig), chaperones, such as *dnj-2(Hsp40)* and *fkb-6(FKBP)*, for which no HLH-1 occupancy site or HLH-1(ec)-induced expression were identified, were unaffected by *hlh-1* knockdown (Fig 4E). Thus, the expression of ubiquitously expressed and muscle-enriched chaperones associated with muscle protein folding and assembly was strongly reduced in *hlh-1(cc561)* embryos.

**Fig 5:**
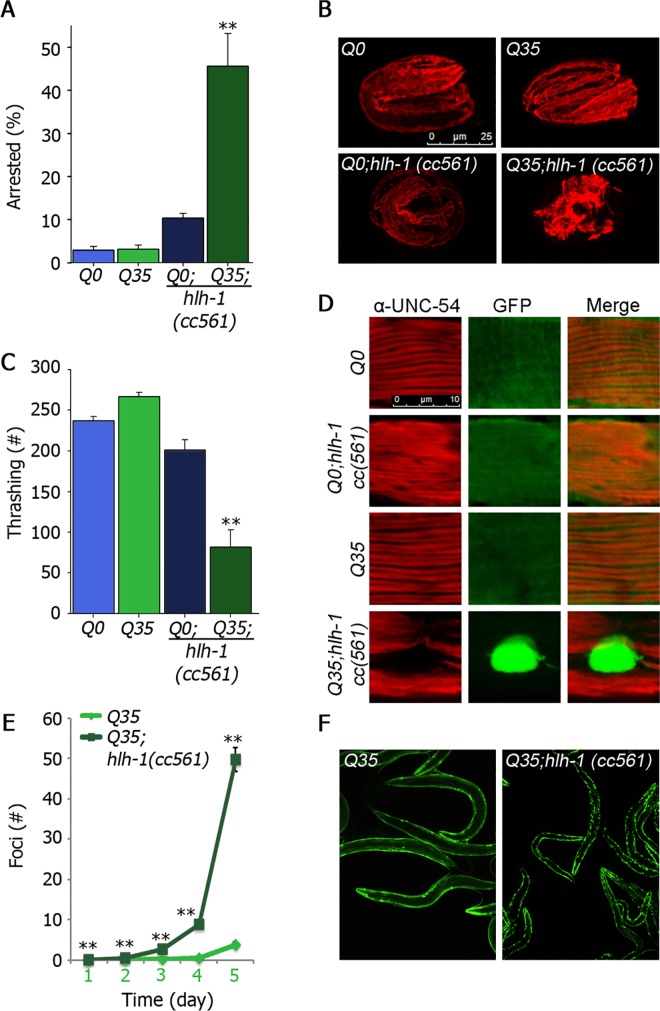
HLH-1 is required for establishing muscle proteostasis. **(A)** Q0, Q35, Q0;*hlh-1(cc561)* or Q35;*hlh-1(cc561)* embryos laid at 15°C were scored for embryonic arrest. Data are presented as means ± SEM of at least 6 independent experiments. **(B)** Representative confocal images of Q0, Q35, Q0;*hlh-1(cc561)* or Q35;*hlh-1(cc561)* embryos laid at 15°C. Scale bar is 25 μm. **(C)** The number of body movements per minute scored in age-synchronized Q0, Q35, Q0;*hlh-1(cc561)* or Q35;*hlh-1(cc561)* animals on the first day of adulthood. **(D)** Representative confocal images of myofilaments. Age-synchronized Q0, Q35, Q0;*hlh-1(cc561)* or Q35;*hlh-1(cc561)* animals expressing GFP (green) and stained with anti-UNC-54 antibodies (red). Scale bar is 10 μm. **(E)** The average number of visible foci scored in age-synchronized Q35 or Q35;*hlh-1(cc561)* animals. **(F)** Images of representative Q35 or Q35;*hlh-1(cc561)* animals 5 days after hatching.

We next considered the consequences of disrupting HLH-1-dependent chaperone expression for muscle proteostasis during embryogenesis. To challenge muscle proteostasis, we crossed *hlh-1(cc561)* with animals expressing yellow fluorescent protein (YFP) fused to 35 glutamine repeats (Q35) or YFP alone (Q0) expressed under the muscle-specific *unc-54* myosin promoter (*Q35;hlh-1(cc561)* and *Q0;hlh-1(cc561)*, respectively). As noted above, *hlh-1(cc561)* is a knockdown mutant. The nonsense allele occurs at a position coding 13 amino acids after the bHLH domain, resulting in a functional protein. Indeed, the *hlh-1(cc561)* phenotype under restrictive conditions was fully rescued by over-expression of the *cc561* allele or by inhibiting the nonsense mRNA decay pathway [57]. Under permissive conditions, <10% of the animals expressing *Q0;hlh-1(cc561)* exhibited embryonic arrest and typical myofilaments were formed (>90%) (Fig 5A and 5B). Likewise, embryonic development was unaffected by Q0- or Q35-expression and myofilament organization, examined by UNC-54 immuno-staining, was normal (Fig 5A and 5B) [11]. In contrast, 45.5±6% of the *Q35;hlh-1(cc561)* embryos were arrested at the two-fold stage and assumed deformed shapes when grown at 15°C. *Q35;hlh-1(cc561)* embryos showed severe mislocalization of UNC-54 and myofilaments were not formed in many of the embryos (>60%, Fig 5A and 5B). This phenotype was partially rescued by inhibiting the nonsense mRNA decay pathway. RNAi knockdown of *smg-2* or *smg-7* did not affect Q35 embryos, yet rescued 30–50% of *Q35;hlh-1(cc561)*-arrested embryos, as compared to those treated with the empty vector control (S5A Fig). Thus, expression of aggregation-prone Q35 in a *hlh-1(cc561)* background resulted in severe disruption of muscle protein folding. These data suggest that muscle proteostasis capacity is limited in *hlh-1(cc561)* embryos, supporting a role for *hlh-1* in establishing muscle proteostasis.

To examine whether reduced HLH-1 levels also impacted muscle proteostasis capacity later in life, i.e., after muscle development has completed, we monitored *Q35;hlh-1(cc561)* young adults for muscle function and myosin organization. Although we excluded deformed or paralyzed animals, motility of *Q35;hlh-1(cc561)* young adults was reduced 2.5-3-fold, as compared to *Q0;hlh-1*, *Q0* or *Q35* young adults (Fig 5C). In agreement, *Q35;hlh-1(cc561)* young adults exhibited severe UNC-54 disorganization, while *Q0;hlh-1(cc561)* myofilaments maintained their striated structures and were only mildly disorganized (Fig 5D and S5B Fig). Myofilament organization was normal in *Q0* or *Q35* young adults (Fig 5D) [11]. Thus, the disruption of muscle protein folding observed for *Q35;hlh-1(cc561)* embryos was not mitigated in adult animals.

Disruption of cellular proteostasis was previously shown to increase Q35 foci formation [11]. Foci formation in *Q35*-expressing animals begins at the transition to reproductive adulthood [58]. As such, no foci were observed in *Q35* animals at the first larval stage (L1) (n = 430). Following the onset of reproduction, (day 5) *Q35* animals had an average of ~4 foci per animal. In contrast, foci were observed in ~10% of the *Q35;hlh-1(cc561)* animals (n = 425) even by the L1 stage, while by day 5, *Q35;hlh-1(cc561)* animals had an average of ~50 foci per animal (Fig 5E and 5F). Still, Q35 protein levels in *Q35;hlh-1(cc561)* animals were ~50% lower than in Q35 animals (S5C and S5D Fig). Thus, reduced HLH-1 levels also resulted in limited muscle proteostasis in adulthood.

The disruption in muscle function and increased aggregation of *Q35;hlh-1(cc561)* later in life could be due to HLH-1 function after embryogenesis but could also stem from defects acquired during myogenesis. Indeed, *Q35;hlh-1(cc561)* L1 animals were already affected at 15°C (Fig 5E). To test the impact of *hlh-1* on proteostasis past embryogenesis, we treated *Q35;hlh-1(cc561)* animals with *smg-2*(*RNAi*) at the L1 stage to rescue *hlh-1* expression levels after embryogenesis was completed. We found that motility and aggregation of *Q35;hlh-1(cc561)* young adults treated with *smg-2*(*RNAi*) from L1 were partially rescued as compared to those treated with the empty vector control (S5E and S5F Fig). In contrast, shifting *hlh-1(cc561)* to 25°C at the L1 stage to reduced *hlh-1* expression levels past embryogenesis, did not significantly affect its motility as compared to wild type (S5G Fig). These data suggest that HLH-1 is not required but can contribute to muscle proteostasis in adulthood. Taken together, our data support a role for HLH-1 in establishing muscle proteostasis, as well as impacting proteostasis capacity in adulthood.

### Modulating muscle chaperone expression can disrupt myogenesis

**Fig 6:**
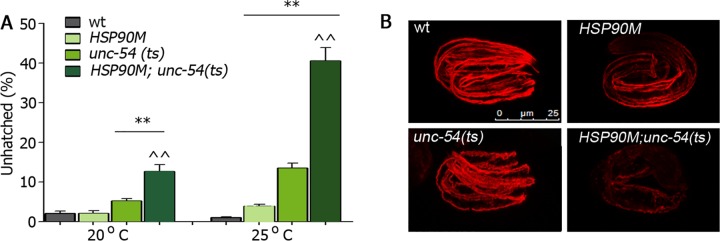
Muscle proteostasis and myogenesis are disrupted in *HSP90M;unc-54(ts)* embryos. **(A)** Wild type, *unc-54(ts)*, *HSP90M* and *HSP90M;unc-54(ts)* embryos laid at the indicated temperature were scored for embryo arrest. Data are presented as means ± SEM of at least 5 independent experiments. **(B)** Representative confocal images (>90%) of wild type, *unc-54(ts)*, *HSP90M* and *HSP90M;unc-54(ts)* embryos laid at 20°C and stained with anti-UNC-54 antibodies. Scale bar is 25 μm.

The correct folding and assembly of myosin thick filaments and thus, myogenesis, requires UNC-45. Myofilaments are assembled and begin to contract some ~420 min after the first division (1.5-fold stage), thereby facilitating embryo elongation (3-fold stage) [34]. In contrast, proper myofilament assembly is disrupted in *unc-45* null mutants, leading to muscle-dependent embryonic arrest at the two-fold stage and lethality. Given that DAF-21(Hsp90) and UNC-45 were shown to compete for myosin binding *in vitro* [59], we postulated that the regulation of ubiquitously expressed chaperone genes by the myogenic transcription factor HLH-1 should also be adjusted to muscle proteomic needs. To directly test whether specifically changing the levels of ubiquitously expressed chaperone in body-wall muscle cells disrupted myogenesis, we asked how over-expression of muscle DAF-21(Hsp90) affected the folding of UNC-54, a known Hsp90 substrate, and hence, myogenesis. A temperature-sensitive mutation in myosin, *unc-54(e1301ts)* (*unc-54(ts)*), shows temperature-dependent misfolding [11] but only mildly induced the arrest at two-fold phenotype [34]. We crossed *unc-54(ts)* with animals that specifically over-express DAF-21(Hsp90) in body-wall muscle cells (strain AM780). These animals express *daf-21(Hsp90)* tagged with GFP (*daf-21*::*GFP*) under the muscle specific *unc-54* promoter (*HSP90M*). We then monitored embryonic arrest and UNC-54 localization in wild type, *HSP90M*, *unc-54(ts)* and *HSP90M;unc-54(ts)* embryos laid at 20 or 25°C. HSP90M did not induce arrest at the two-fold stage when the animals were grown at 20 or 25°C (2.1±0.6% and 3.9±0.5%, respectively). *unc-54(ts)* embryos showed a mild arrest at 20 and 25°C (5.2±0.6% and 13.6±1.2%, respectively). In contrast, *HSP90M;unc-54(ts)* embryos were severely delayed (S6A Fig), with the percentage of embryo arrested at the two-fold stage at both 20 and 25°C being increased (12.7±1.7% and 40.6±3.3%, respectively, Fig 6A). *HSP90M;unc-54(ts)* embryos showed defective myofilament and muscle elongation. Immuno-staining with anti-UNC-54 antibodies of *HSP90M;unc-54(ts)* embryos grown at 20°C exhibited strongly reduced UNC-54 staining (Fig 6B). Although the embryos examined were arrested at the two-fold stage, most eventually hatched (Fig 6A). Similar UNC-54 immuno-staining was observed for *HSP90M;unc-54(ts)* embryos grown at 25°C but only about half of these embryos hatched (Fig 6A and S6B Fig). In contrast, UNC-54 myofilament assembled correctly in most wild type, *HSP90M*, *unc-54(ts)* embryos grown at 20°C (Fig 6B). Our data suggest that DAF-21(Hsp90) levels are adjusted for proper myosin folding to support muscle elongation and embryo development. Thus, changes in chaperone expression can disrupt proteostasis and abrogate myogenesis.

### Modulating chaperone expression disrupts the chaperone network

**Fig 7:**
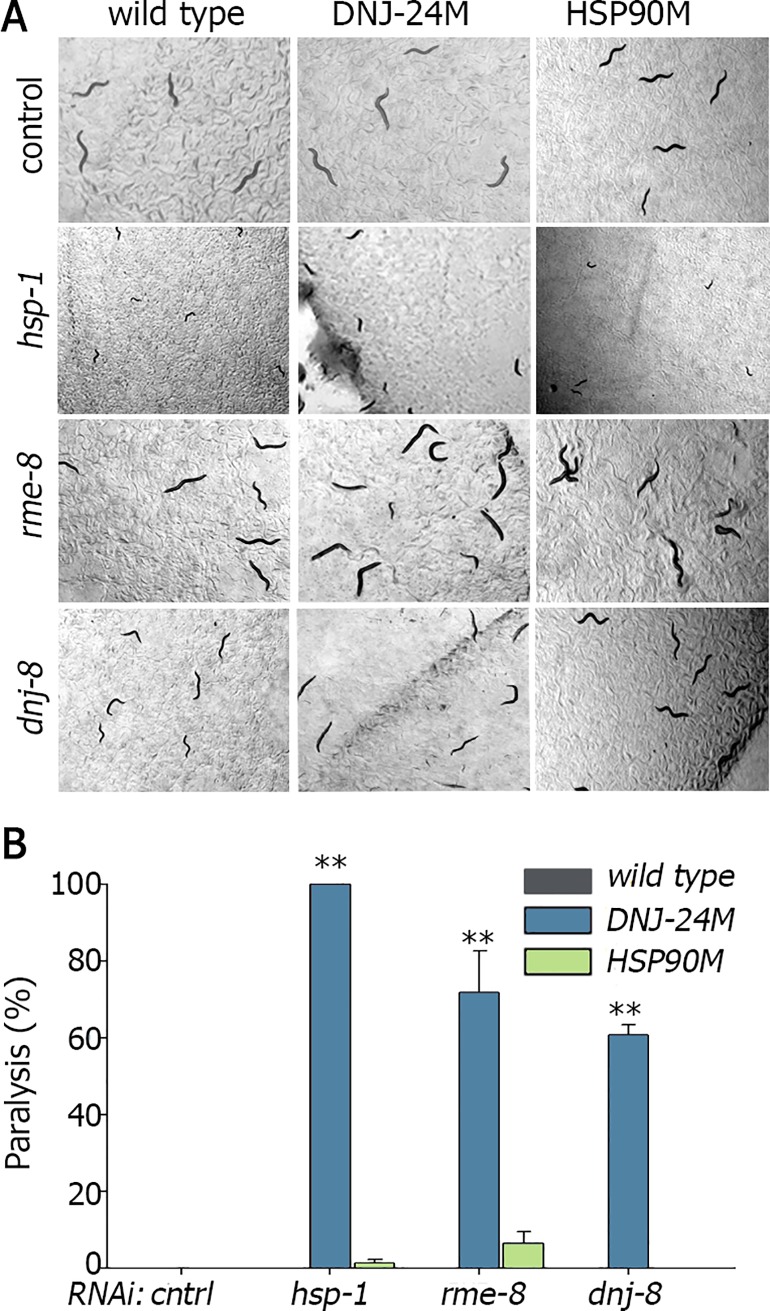
Muscle over-expression of *dnj-24* disrupts chaperone interactions, exposing sensitivity to specific chaperone down-regulation. **(A)** Age-synchronized L1 wild type, DNJ-24M or HSP90M animals grown at 15°C were transferred to plates containing control, *hsp-1*, *rme-8*, or *dnj-8* RNAi-expressing bacteria, and images were taken on day 1 of adulthood. **(B)** Age-synchronized wild type, DNJ-24M or HSP90M animals treated as in (A) were scored for motility on day 1 of adulthood. Data are presented as means ± SEM of 3 independent experiments.

The expression of aggregation-prone proteins was suggested to disrupt proteostasis by engaging chaperones and competing for their substrates [9, 11]. Differences in chaperones expression levels and composition could also alter chaperone and co-chaperone interactions. Thus, modulating chaperone expression in a given tissue could transform the network of that chaperone. To ask how changing chaperone levels modulate chaperone interactions, we focused on *dnj-24(Hsp40)*, encoding the *C*. *elegans* homolog of DNAJB6. DNAJB6 is a ubiquitously expressed chaperone linked to limb-girdle muscular dystropy type 1D (LGMD1D) [18]. LGMD1D mutations were shown to result in stabilization and, therefore, increased levels of DNAJB6. While the amino acids associated with LGMD1D are not conserved in DNJ-24(Hsp40), DNJ-24(Hsp40) is enriched in muscle and shows the expected muscle and nuclear distribution pattern [49]. To address whether increased levels of this chaperones disrupted chaperone interactions in muscle cells, we examined the effects of muscle over-expression of *dnj-24(Hsp40)* (DNJ-24M) on synthetic motility defects induced by chaperone knock-down. We reasoned that if DNJ-24M perturbed chaperone interactions in muscle cells, then this might exacerbate the effects of knocking-down the levels of other muscle chaperones [25]. If so, then RNAi of chaperones that do not affect motility in wild type animals should induce motility defects in DNJ-24M-expressing animals. Consistent with previous work in a zebrafish model [18], over-expression of wild type *dnj-24(Hsp40)* in body-wall muscle of *C*. *elegans* did not result in notable motility defects (Fig 7). However, when age-synchronized DNJ-24M-expressing animals were treated with RNAi for different Hsp70 chaperones and co-chaperones, three genes (of 48 examined), namely *hsp-1*, *rme-8*, and *dnj-8*, specifically affected the motility of DNJ-24M-expressing but not wild type or HSP90M-expressing animals (Fig 7A and 7B). RNAi knock-down of the *hsp-1(Hsc70)* induced a strong larval arrest in wild type, HSP90M and DNJ-24M animals, yet only in the DNJ-24M animals did such treatment induce 100% paralysis (Fig 7A and 7B). Of note, DNAJB6 interacts with several chaperones associated with chaperones-assisted selective autophagy, one of which is HSPA8, a Hsp-1(Hsc70) homolog [18]. Knocking-down the expression of *rme-8(Hsp40)* and *dnj-8(Hsp40)* resulted in no motility phenotype in wild type or HSP90M animals, while knocking-down the expression of these genes in a DNJ-24M background resulted in motility defects (72±10.7 and 61±2.7, p<0.0002, Fig 7B) and disrupted myosin organization (S7 Fig). Thus, over-expression of DNJ-24(Hsp40) in body-wall muscle cells disrupted muscle proteostasis such that muscle cells were more susceptible to *hsp-1(Hsc70)*, *rme-8(Hsp40)* and *dnj-8(Hsp40)* knock-down. Cell type-specific regulation of chaperone expression could, therefore, impact tissue-specific chaperone networks.

## Discussion

### Differentiation can establish cellular proteostasis

In the present study, we asked whether the cellular chaperone network is regulated in a cell type-specific manner. Specifically, we asked whether muscle chaperones are regulated by the myogenic transcription factor HLH-1 during *C*. *elegans* myogenesis. We found that muscle chaperones that have HLH-1 occupancy sites in their promoter are induced in myogenic-converted embryos. This muscle-specific induction was fully dependent on HLH-1, as no induction was observed for most chaperones without HLH-1 occupancy sites or when HLH-1 expression was down-regulated. Moreover, we showed that disrupting the putative HLH-1 binding sites in two different chaperone promoters inhibited their myogenic-induced expression and muscle expression later in life. Thus, HLH-1 is required for the expression of muscle chaperones with HLH-1 occupancy sites in cells undergoing differentiation into body-wall muscle cells. While a HLH-1 differentiation-independent function in embryonic muscle cells is possible, we instead propose that muscle chaperone genes are regulated by HLH-1 together with other muscle genes during myogenesis. Linking the regulation of chaperone expression to the differentiation program could result in a distinct chaperone network, ensuring that chaperones are expressed at the required levels and with proper timing. Indeed, we found that down-regulation of HLH-1 strongly restricted proteostasis capacity, leading to misfolding of muscle protein and myogenesis arrest.

Tissue-specific differences in the expression levels of chaperones can explain why down-regulation of ubiquitously expressed chaperones led to a tissue-selective activation of the heat shock response [60] and why the cellular folding environment is sensitive to chronic expression of aggregation-prone proteins and expression of stress-induced chaperones [9, 11, 19, 61]. The importance of regulating chaperone levels in a tissue-specific manner is supported by prior findings and our data showing that both down-regulation and over-expression of the myosin-specific chaperone UNC-45 and the ubiquitously expressed Hsp90 were detrimental to myosin assembly and muscle elongation [45, 59]. Given that DAF-21(Hsp90) and UNC-45 were shown to compete for myosin binding *in vitro*, their relative levels are critical for myosin folding and can abrogate myogenesis. We, therefore, suggest that physiological tissue-specific chaperone networks can enable cells to respond to the folding requirement of their unique proteomes, leading to distinct responses to folding challenges, such as acute stress or chronic expression of misfolded proteins.

We found that muscle proteostasis can also be critical for muscle differentiation and can, as in the case of UNC-45 and Hsp90, lead to embryonic arrest and lethality. In support of this claim, a recent study showed that activation of Janus kinase 2 (JAK2) signaling associated with myeloproliferative neoplasms (MPNs) resulted in reduced expression of proteostasis component AIRAPL (arsenite-inducible RNA-associated protein-like) and led to increased insulin/insulin-like growth factor 1 (IGF1R) stability and disrupted hematopoietic differentiation [62]. Thus, changes in expression of proteostasis components can result in modulated folding or degradation of cellular factors, such as signaling proteins that, in turn, can lead to alterations in differentiation.

### A general role for differentiation-related transcription factors in regulating cell type-specific proteostasis

Our data demonstrate that the myogenic factor HLH-1 regulates the expression of chaperones in muscle cells. By extension, chaperones can be differentially regulated in different cell types to meet the needs of a specific proteome. The large available ChIP-seq dataset (ModEncode) [63] shows that transcription factors involved in development and differentiation can bind the promoter region of chaperone genes, supporting our proposal of tissue-specific developmental regulation of chaperone expression, and raises the possibility that tissue differentiation promotes the expression of required chaperones. In agreement, PHA-4 is required for the development of the pharynx and foregut and also regulates the expression of autophagy-required genes [64]. ChIP-seq analysis of the occupancy sites of PHA-4 [65] showed significant overlap between PHA-4 binding to chaperone promoters under starvation stress conditions and chaperone promoters occupied during embryogenesis (33 out of 38 overlapped between L1 stress and embryos, p = 0.0001). This overlap suggests, in turn, a possible role for transcription factors involved in development and differentiation in tissue maintenance later in life. Indeed, the *pha-4* occupancy site in the *daf-21(hsp90)* promoter, as identified by modEncode, was shown to be functional in cell non-autonomous expression of *daf-21(Hsp90)* in adult *C*. *elegans* muscle, intestinal and neuronal cells [12]. For HLH-1, we observed disrupted proteostasis in adulthood in a Q35;*hlh-1(cc561)* background that could be alleviated by blocking the nonsense mRNA decay pathway and thus, *hlh-1* mRNA clearance. Indeed, *hlh-1(cc561)* exacerbated the effect of a dystrophin mutation associated with Duchenne’s muscular dystrophy, leading to muscle degeneration in adulthood [66, 67]. We, therefore, propose that similar to the specialization of the chaperone networks in unicellular eukaryotes into two separate sets, one dedicated to coping with stress-induced misfolding and the other to newly translated proteins [26], the regulation of chaperone expression in multi-cellular organisms is specialized to establish chaperone networks dedicated to the folding and maintenance of cell type-specific proteomes in development and possibility in adulthood as well.

Myogenic-dependent regulation of chaperones suggests that the proteostatic requirements of the muscle proteome might dictate the expression of other quality control machineries to fit functional and folding characteristics of that proteome. Careful analysis of HLH-1 targets revealed that most were not muscle-enriched [30]. One interpretation of this analysis is that the expression of general factors required for muscle differentiation is specifically regulated to meet the needs of muscle cells. Indeed, SKN-1, required for specification of the EMS blastomeres that give rise to pharyngeal, muscle and intestinal cells, regulates the expression of the oxidative stress response [68]. As noted above, PHA-4 is involved in both development and autophagy [64]. Moreover, autophagy is activated in different tissues of zebrafish during embryogenesis and is required for vertebrate cardiac morphogenesis [69]. Likewise, efficient differentiation of human embryonic stem cells required increased expression of the 19S subunit PSMD11 [70]. We, therefore, propose that rather than relying on a generic proteostatic machinery, each cell and tissue type with a defined folding capacity possesses a specific composition of the quality control machinery and perhaps even cell type-specific heat shock response and unfolded proteins responses to deal with the highly specialized challenges of each cell type.

### Implications of cell type-specific regulation for misfolding diseases

Although the expression of many disease-associated proteins is not tissue-specific, many protein misfolding diseases exhibit tissue-specific vulnerability [71, 72]. For example, mutations in the ubiquitously expressed co-chaperone DNAJB6 cause a tissue-specific disease, limb-girdle muscular dystropy [18]. The mechanism for this selective vulnerability in certain tissues is unknown, although differences in folding and clearance capacities were suggested to affect the onset of several tissue-specific diseases and stress activation [60, 73–75]. Here, we showed that over-expression of the DNAJB6 homolog DNA-24(Hsp40) in muscle cells affected the muscle function of HSP-1(Hsc70) and two other DnaJ co-chaperones, RME-8(Hsp40) and DNJ-8(Hsp40). HSP-1(Hsc70) and RME-8(Hsp40) are required for receptor-mediated and fluid-phase endocytosis [76, 77]. This suggests that the balance between co-chaperones may affect Hsc70 function, similar to protein misfolding [9, 11]. Indeed, a lack of RME-8(Hsp40) resulted in mislocalization and clearance of endosomal proteins to the lysosome, associated with autophagic function [78]. Given the link of DNAJB6 to autophagy [18], it is possible that DNJ-24M disrupts HSP-1(Hsc70) and RME-8(Hsp40) interactions, in turn affecting endocytic trafficking in LGMD1D. Thus, the observed stabilization of DNAJB6 might play a role in LGMD1D muscle etiology by competing for RME-8(Hsp40) or other Hsp40s function. Differential susceptibility to misfolding or stress may, therefore, spring from cell-specific differences in the composition and expression levels of components of the proteostatic network. We propose that differences in chaperone levels and composition between tissues could impact tissue-specific vulnerability to protein misfolding diseases that are globally expressed yet which are manifested in a specific tissue.

## Methods

### Bioinformatics and statistics

The chaperone list was complied based on the work of Brehme *et al*. [79], focusing on the main chaperone families and their co-chaperones (97 genes), including Hsp60 and Hsp10, Hsp70, Hsp40 and NEF, Hsp90 and Hsp90 co-chaperones and sHSP [25]. Three curated lists of HLH-1 occupancy sites were used, i.e. ChiP-seq (e^-6^) peak call data, were provided in the manuscript as supporting information [29] and two curated lists, namely a union set of genes that were identified in experiment (*mex-3* or *mex-3;skn-1;elt-1* RNAi) and an overlap set identified in both [30]. The later curated lists (Union and overlap) were kindly provided by Dr. Steven Kuntz and Dr. Paul Sternberg (S1 Table). Three curated lists of genes enriched in muscle were used: (1) Myogenic-converted embryos [30], kindly provided by Dr. Steven Kuntz and Dr. Paul Sternberg; (2) Muscle cells from dissociated embryos and (3) L1 body-wall muscles [31, 40]. These later curated lists (2–3) were provided in the manuscripts as supporting information. Chaperone genes occupancy sites and muscle enrichment were ranked according to the number of independent experiments in which they were identified, giving equal weight to each experiment. Muscle-required chaperone genes were ranked according to the number of criteria (function, phenotype or sarcomeric localization) they fulfilled. The list was sorted by HLH-1 occupancy ranking (Fig 1A). The flowchart outlining the bioinformatics analyses and all the data included in these analyses are summarized in S1 Table.

HLH-1 binds to E-Box motif (CANNTG) [29, 80]. For each of the 97 chaperone genes, we downloaded upstream sequences (1000bp) from the ensambel biomart webserver and searched using the FIMO tool from MEME suite 4.11.2 with a p-value<0.001 [81]. Putative HLH-1 E-box-binding motifs were found at the promoters of 39 of the 62 chaperones with HLH-1 occupancy sites but were not enriched in these promoters (S1 Table).

Venn diagrams were plotted using the BioVenn diagram generator http://www.cmbi.ru.nl/cdd/biovenn/ (BioVenn) [82]. Microarray-normalized data for *C*. *elegans* embryonic development gene expression was provided by Dr. Itai Yanai [55]. Data were complied and clustered using the EXPANDER (6.5.1) program [83]. The probability of overlap between chaperone sets was calculated using the Fisher exact test. The *P* values in Figs 2–7 were calculated using the Mann-Whitney test, where (*) denotes P<0.05 and (**) denotes P<0.01.

### Nematode strains and maintenance

The list of strains used in this work is provided in S2 Table. Nematodes were grown on NGM plates seeded with the *Escherichia coli* OP50-1 strain at 15°C, unless indicated otherwise. Cross-strains were generated using standard *C*. *elegans* procedures.

To generate the promoter reporter constructs for *daf-21(Hsp90)* and *hsp-12*.*2(sHsp)*, a 2492 bp fragment for *daf-21(Hsp90)* and a 921 bp fragment for the *Hsp-12*.*2(sHsp)* promoter were amplified from N2 genomic DNA and assembled into plasmid pNU106 to create plasmids pNU314 and pNU374, respectively, using Gibson ligation. Mutated promoter reporters for *daf-21(Hsp90)* and *hsp-12*.*2(sHsp)*, *pNU315*::*Pdaf-21(mut)*::*gfp* and *pNU375*::*hsp-12*.*2p(mut)*::*gfp*, respectively, were generated by site-directed mutagenesis of the putative HLH-1-binding motifs at -432 bp and at -921 bp and -266 bp to ACGCGT. Plasmids were validated by DNA sequencing and injected into animals expressing *unc-119(ed3);hsp-16*.*41*::*hlh-1*. Promoter reporter constructs were generated and injected by Knudra Transgenics. Stable transgenic lines are listed in S2 Table.

### Embryo synchronization and treatment

Synchronized animals were grown at 15°C for five days or transferred to 20 or 25°C at the L2 stage for 24–48 h to reach adulthood. These synchronized gravid adults (first day of egg laying) were allowed to lay eggs for 45 min and then removed. Synchronized embryos were heat shock-treated, allowed to grow for 6 h to pass the comma stage or allowed to grow for 24–48 h and complete embryogenesis.

### Heat shock treatment

Embryos laid at 15°C were moved to new plates and were untreated or subjected to heat shock at 34°C for 30 min. To determine RNA levels, embryos were frozen immediately following heat shock. To examine expression patterns, embryos were collected after a 6 h recovery.

### Temperature shift treatment

Wild type and *hlh-1(cc561)* embryos laid at 15°C or 25°C for 45 min were allowed to grow for 6 h. The embryonic developmental stage was examined to determine whether embryos grown at 15°C had passed the comma stage. To determine the effect of *hlh-1(cc561)* on gene expression, we compared the relative mRNA levels (25°C/15°C) of the genes examined. In S5G Fig, the temperature shift was carried out at L1.

### RNA interference experiments

RNAi knockdown treatments were performed as previously described [84]. RNAi constructs were obtained from the “RNAi chaperone library” kindly provided by Prof. Richard Morimoto, Northwestern University [79] or from the Julie Ahringer library. To collect RNAi-treated embryos, animals were grown on *E*. *coli* strain HT115(DE3) transformed with specified RNAi or empty (pL4440) vectors and allowed to lay eggs, as above. Otherwise, synchronized L1 larvae stage worms were washed from regular NGM plates, transferred to RNAi plates and grown at 15°C for five days until day 1 of adulthood.

### RNA levels

RNA extraction from synchronized embryos, cDNA synthesis and quantitative real-time PCR were performed as previously described [25]. Samples were normalized (2^-ΔΔC^_T_ method) to *T07A9*.*15* or *tbc-10*, determined to be stably expressed during embryogenesis [55]. The list of primers used in this work is provided in S3 Table.

### Embryo arrest

To determine embryo arrest, synchronized embryos were grown at 15–25°C. Embryos that did not hatch until their siblings reached L2 (24–48 hours) or hatched arrested at the two-fold state were counted as arrested embryos.

### Motility assays

Age-synchronized young adults were moved to a new plate and their movement was monitored after 10 min. Animals that did not move one body length were scored as paralyzed. Otherwise, age-synchronized young adults were placed in wells containing M9 and allowed to acclimate for 5–10 min. Each animal was monitored for 15 sec and thrashes (changes in direction of bending at mid-body) were counted.

### Immuno-staining and fluorescence reporters

Embryos were fixed in methanol and 2% paraformaldehyde and permeabilized by freeze-thawing and then immuo-stained as described [85]. Adult animals were fixed with 4% paraformaldehyde and permeabilized with β-mercaptoethanol and collagenase IV treatment as described [86]. Antibodies used in this work included anti-MYO-3 (5–6), anti-UNC-45 (gift from Dr. Thorsten Hoppe) and anti-UNC-54 (28.2, gift from Dr. Jose Barral) [87, 88] and secondary DyLight 488, DyLight 549 or DyLight 633 anti-mouse or anti-rabbit antibodies (Jackson Immuno Research). Embryos or adult animals expressing fluorescence reporters or tagged proteins were fixed with 4% paraformaldehyde. Treated samples were imaged using an Olympus Fluoview FV1000 or an LEICA DM5500 confocal microscope with 488 or 549 or 633 nm laser lines for excitation or with LEICA DFC360FX camera. Otherwise, treated samples were imaged using an LEICA M165FC stereomicroscope with QIMAGINE Exi blue camera.

### Aggregation quantification

The number of bright foci of age-synchronized animals expressing Q35::YFP was counted using an LEICA M165FC stereomicroscope.

### Protein levels

Age-synchronized animals were collected and lyzed in SDS sample buffer (95°C for 10 min). Samples were separated by SDS-PAGE and analyzed by western blot (LF PVDF membrane), using primary anti-tubulin (Sigma) and anti-GFP (Enco Scientific) and secondary DyLight 488 and DyLight 549, anti-mouse and anti-rabbit antibodies, respectively (Jackson Immuno Research). Membranes were imaged using the ChemiDoc MP Imaging System (BioRad).

## Supporting Information

S1 Fig Transcriptional analysis of muscle gene expression. Hierarchical clustering of the relative expression of 35 muscle-specific genes across 10 developmental stages (at 4-cells, E cell division, 4^th^-7^th^ AB cell divisions, ventral enclosure (VE), comma stage (cs), first movement, and L1) [55]. MI marks the myogenesis-induced subset.
(TIF)

S2 Fig Heat shock-induced changes in chaperone expression. **(A)** Representative images (>90%) of the expression pattern of chaperones in wild type embryos untreated or subjected to heat shock after a 6 h recovery. Scale bar is 25 μm. **(B-E)** Relative chaperone mRNA levels in heat shock-treated wild type (gray) or HLH-1(ec) (red) embryos. Data are relative to values obtained with untreated embryos (normalized to *tbc-10*) and are presented as means ± SEM of at least 5 independent experiments.
(TIF)

S3 Fig A mutation in the putative HLH-1-binding motif of *daf-21(Hsp90)* promoter affected its expression pattern in adult animals. Representative images of HLH-1(ec) animals expressing GFP under the regulation of the wild type or a mutant *daf-21(Hsp90)* promoter, without myogenic induction. Arrows indicate body-wall muscle cells.
(TIF)

S4 Fig Reduced HLH-1 levels modulate the expression of some chaperones. **(A)** Representative images (>90%) of the expression pattern of the indicated chaperones in wild type embryos grown at 25°C. **(B-C)** Relative mRNA levels (25/15°C) of wild type (gray) or *hlh-1(cc561)* (green) embryos (normalize to *T07A9*.*15*). Data are presented as means ± SEM of 5 independent experiments.
(TIF)

S5 Fig Disruption of muscle proteostasis results in embryo arrest. **(A)** Embryonic arrest scored for Q35;*hlh-1(cc561)* or Q35 embryos treated with *smg-2*, *smg-7* or empty vector control RNAi. Data are presented as means ± SEM of at least 3 independent experiments. **(B)** Representative confocal images of Q35;*hlh-1(cc561)* muscles. Scale bar is 10 μm. **(C-D)** Extracts of age-synchronized (day 4) *Q35* or Q35;*hlh-1(cc561)* animals were separated on a SDS-PAGE gel and probed with anti-GFP (top) and anti-tubulin (bottom) antibodies. Relative levels were determined by quantification of Q35::YFP protein bands. Data are presented as means ± SEM of at least 3 independent experiments. **(E)** The number of body movements per minute scored on the first day of adulthood in age-synchronized Q35;*hlh-1(cc561)* animals treated with *smg-2* or empty vector control RNAi from L1. **(F)** The average number of visible foci scored in age-synchronized Q35;*hlh-1(cc561)* young adults treated with *smg-2* or empty vector control RNAi from L1. **(G)** The number of body movements per minute scored in wild type or *hlh-1(cc561)* young adults shifted to 25°C at L1.
(TIF)

S6 Fig Modulating HSP90 levels results in embryo arrest. **(A)** Images of a population of wild type, *unc-54(ts)*, HSP90M or HSP90M;*unc-54(ts)* embryos laid at 20°C. **(B)** Representative confocal images (>90%) of *unc-54(ts)*, and *HSP90M;unc-54(ts)* embryos laid at 25°C and stained with anti-UNC-54 antibodies. The scale bar is 25 μm.
(TIF)

S7 Fig Down-regulation of *hsp-1(Hsc70)*, *rme-8(Hsp40)* and *dnj-8(Hsp40)* in DNJ-24M animals disrupts myosin organization. Representative confocal images of age-synchronized DNJ-24M animals treated with control, *hsp-1(Hsc70)*, *rme-8(Hsp40)* or *dnj-8(Hsp40)* RNAi and stained with anti-MYO-3 antibodies. Scale bar is 25 μm.
(TIF)

S1 Table Chaperone association with muscle and HLH-1 binding. **(A)** Flowchart outlining the filtering analyses of the chaperone list. **(B)** Summarized data from bioinformatics analyses.
(XLSX)

S2 Table List of strains used in this study. (PDF)

S3 Table List of quantitative PCR primers used in this study. (PDF)
