# Supplementary figures and images for: A Differentiation Transcription Factor Establishes Muscle-Specific Proteostasis in Caenorhabditis elegans
Source: PLoS Genet. 2016 Dec 30;12(12):e1006531. doi: 10.1371/journal.pgen.1006531 (PMC5201269; doi:10.1371/journal.pgen.1006531)

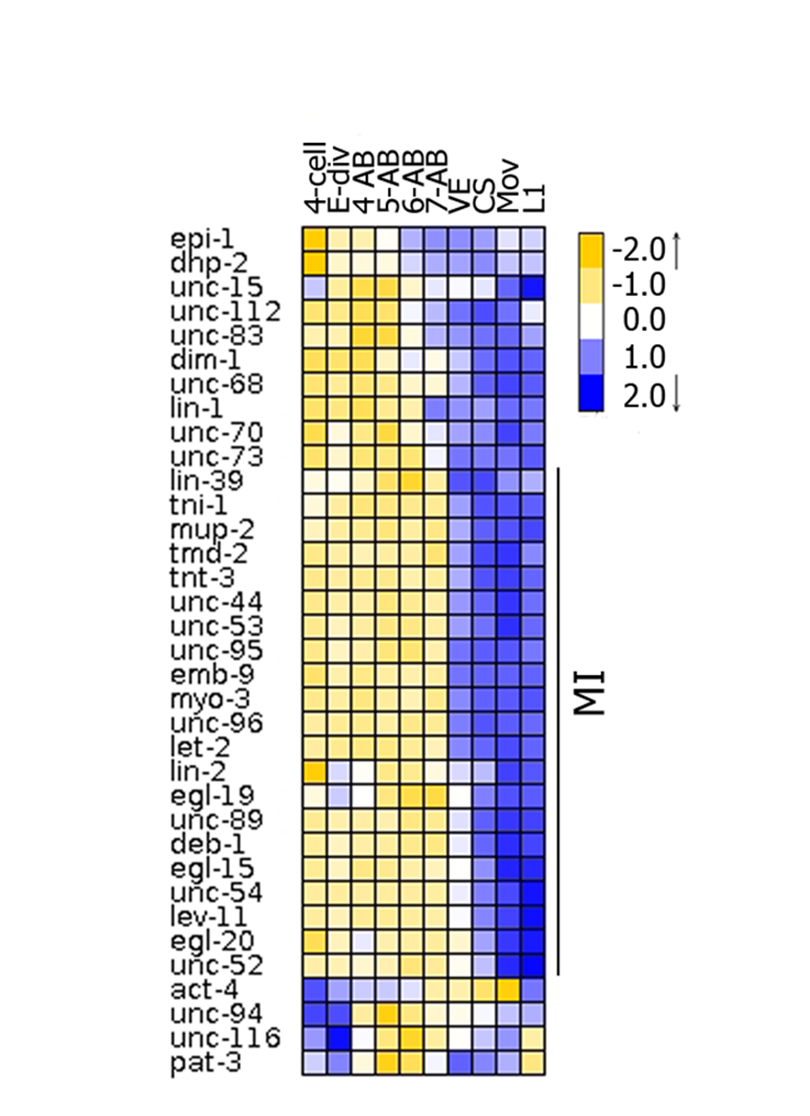

Supplement: S1 Fig — Hierarchical clustering of the relative expression of 35 muscle-specific genes across 10 developmental stages (at 4-cells, E cell division, 4th-7th AB cell divisions, ventral enclosure (VE), comma stage (cs), first movement, and L1) [55]. MI marks the myogenesis-induced subset. (TIF) [file pgen.1006531.s001.tif]

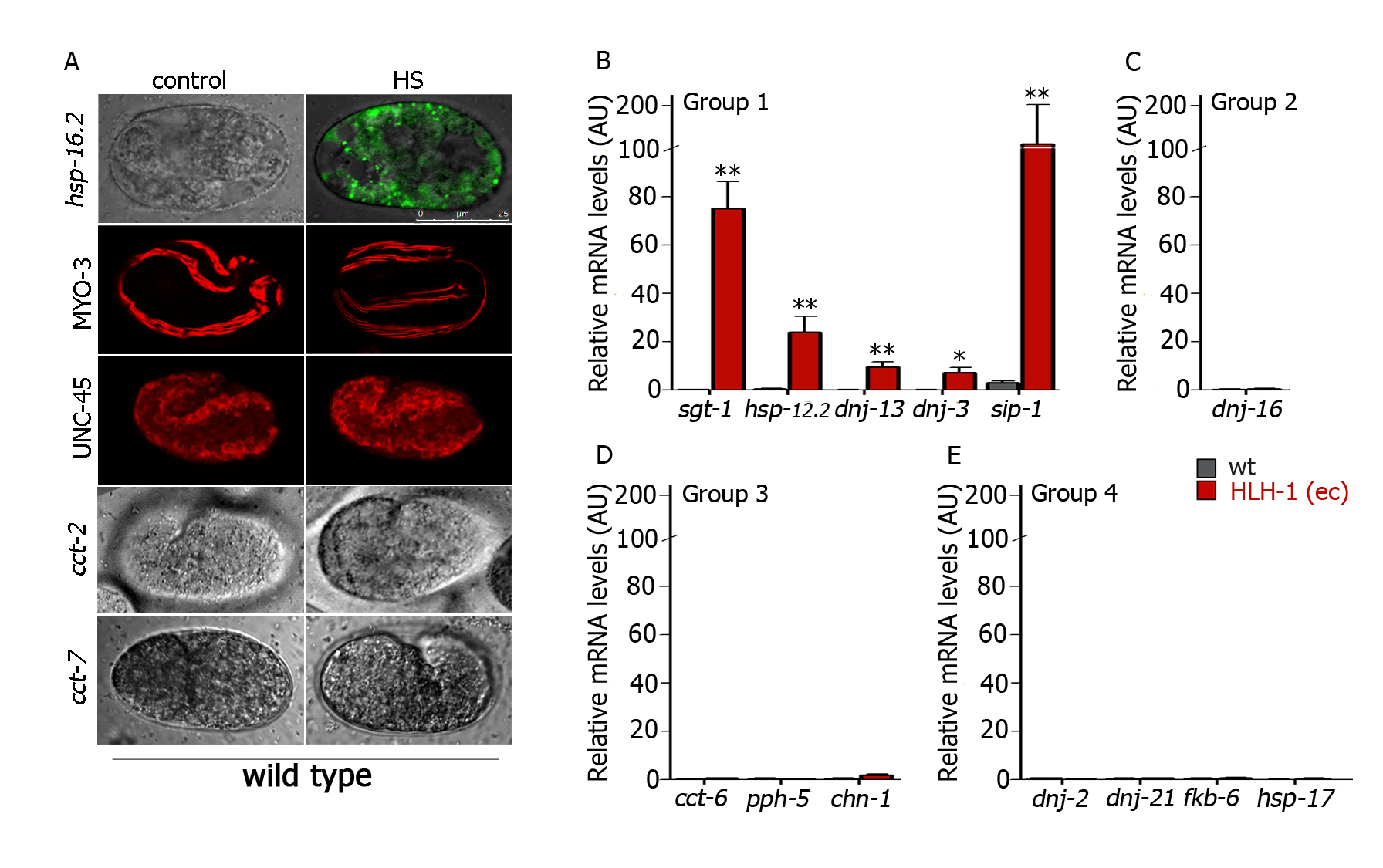

Supplement: S2 Fig — (A) Representative images (>90%) of the expression pattern of chaperones in wild type embryos untreated or subjected to heat shock after a 6 h recovery. Scale bar is 25 μm. (B-E) Relative chaperone mRNA levels in heat shock-treated wild type (gray) or HLH-1(ec) (red) embryos. Data are relative to values obtained with untreated embryos (normalized to tbc-10) and are presented as means ± SEM of at least 5 independent experiments. (TIF) [file pgen.1006531.s002.tif]

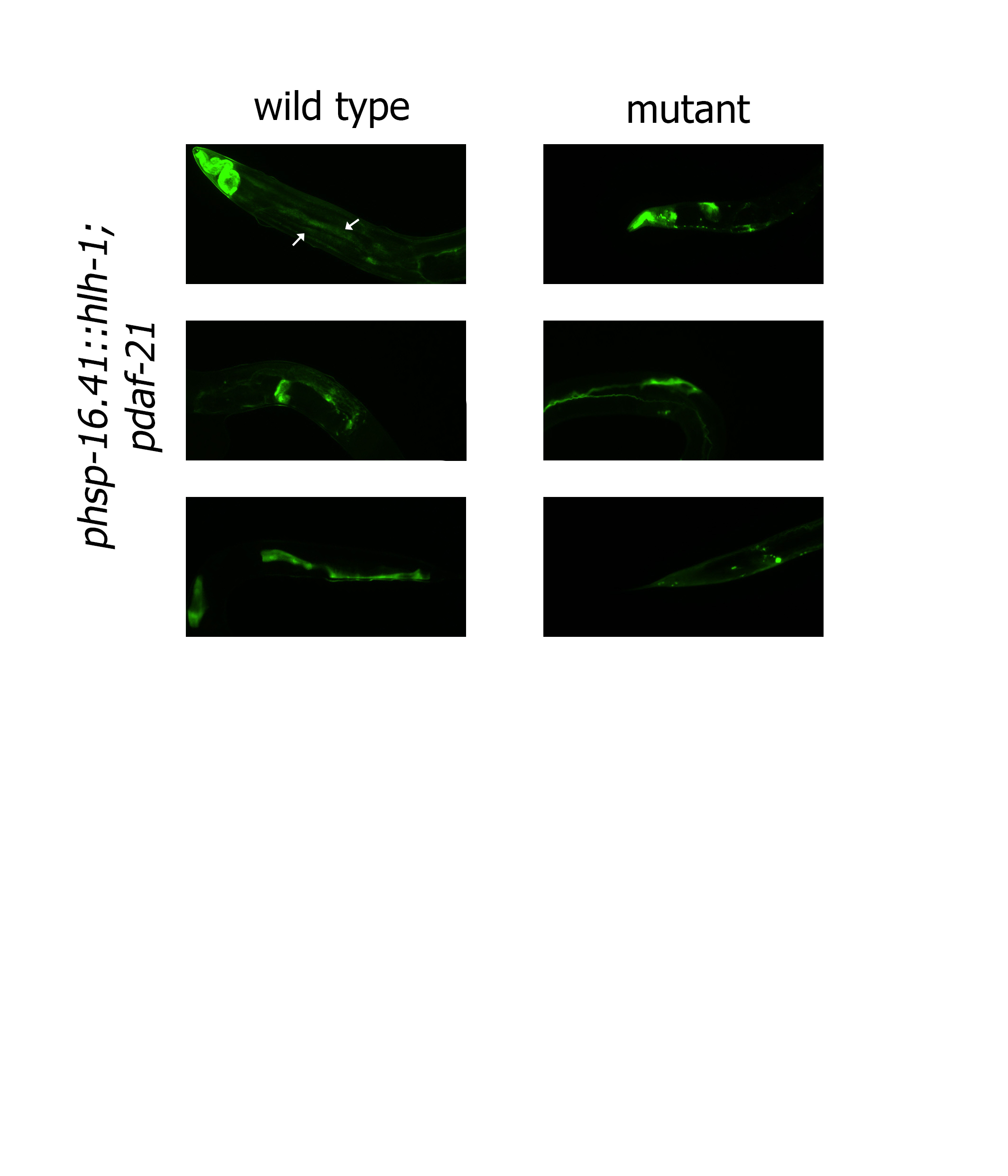

Supplement: S3 Fig — Representative images of HLH-1(ec) animals expressing GFP under the regulation of the wild type or a mutant daf-21(Hsp90) promoter, without myogenic induction. Arrows indicate body-wall muscle cells. (TIF) [file pgen.1006531.s003.tif]

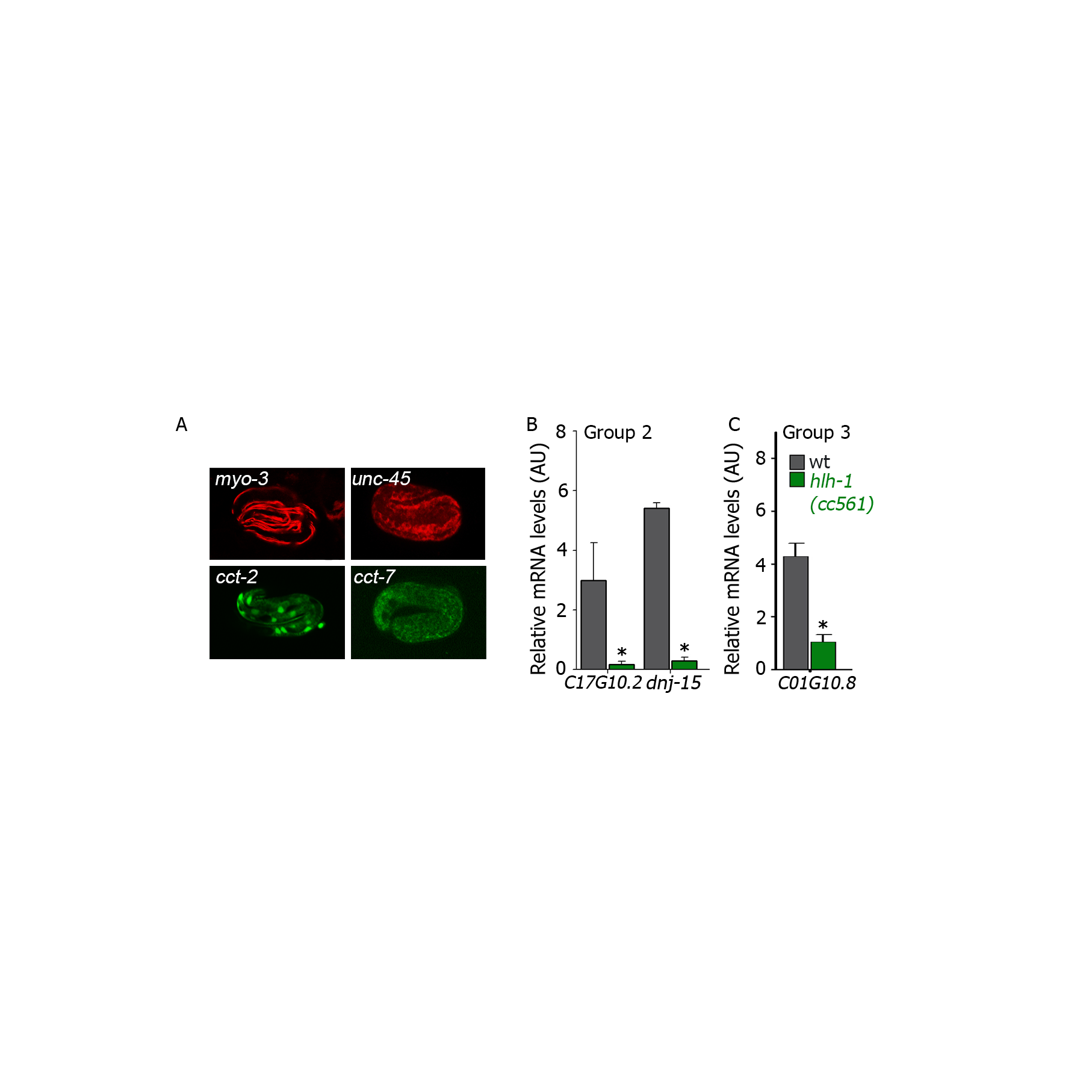

Supplement: S4 Fig — (A) Representative images (>90%) of the expression pattern of the indicated chaperones in wild type embryos grown at 25°C. (B-C) Relative mRNA levels (25/15°C) of wild type (gray) or hlh-1(cc561) (green) embryos (normalize to T07A9.15). Data are presented as means ± SEM of 5 independent experiments. (TIF) [file pgen.1006531.s004.tif]

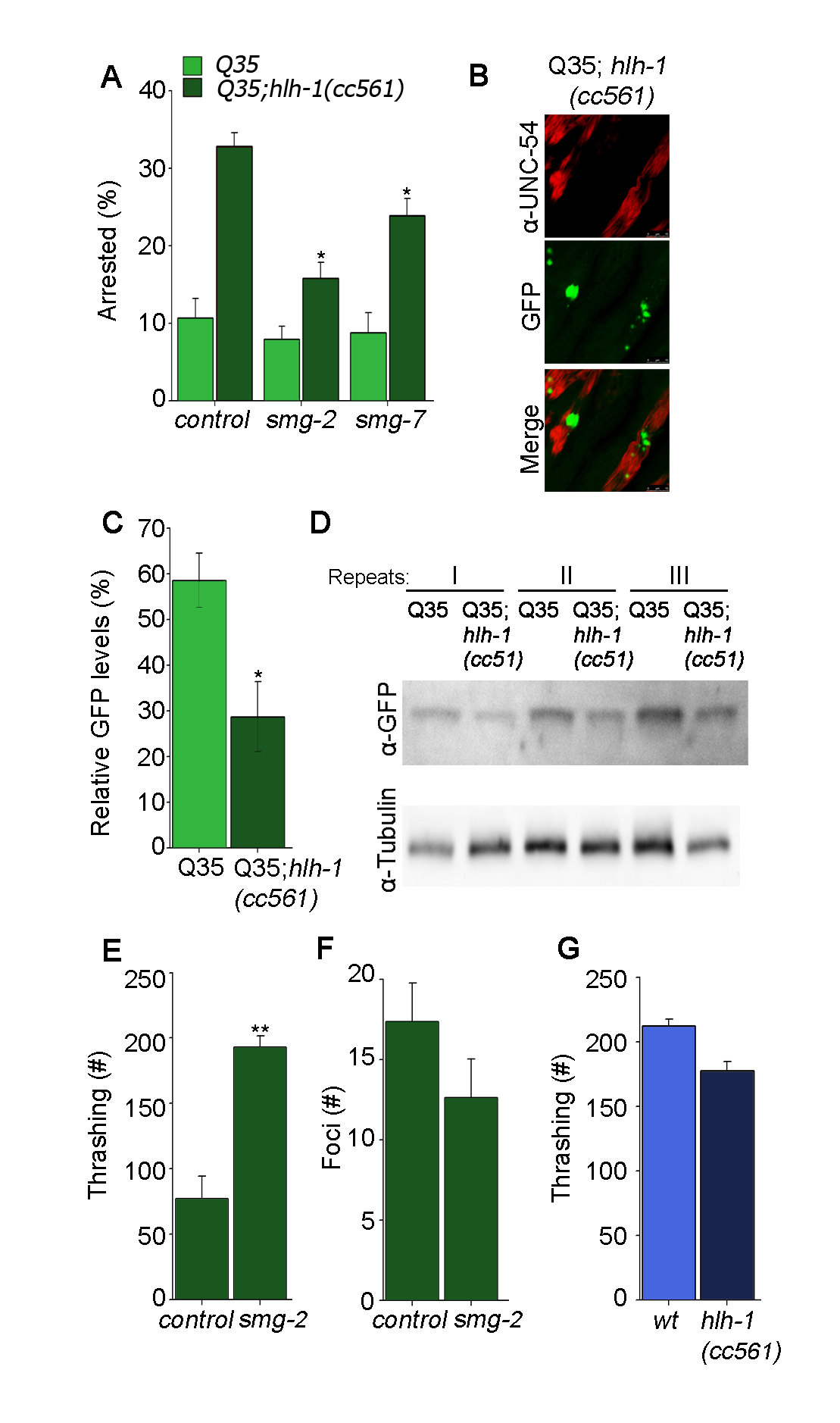

Supplement: S5 Fig — (A) Embryonic arrest scored for Q35;hlh-1(cc561) or Q35 embryos treated with smg-2, smg-7 or empty vector control RNAi. Data are presented as means ± SEM of at least 3 independent experiments. (B) Representative confocal images of Q35;hlh-1(cc561) muscles. Scale bar is 10 μm. (C-D) Extracts of age-synchronized (day 4) Q35 or Q35;hlh-1(cc561) animals were separated on a SDS-PAGE gel and probed with anti-GFP (top) and anti-tubulin (bottom) antibodies. Relative levels were determined by quantification of Q35::YFP protein bands. Data are presented as means ± SEM of at least 3 independent experiments. (E) The number of body movements per minute scored on the first day of adulthood in age-synchronized Q35;hlh-1(cc561) animals treated with smg-2 or empty vector control RNAi from L1. (F) The average number of visible foci scored in age-synchronized Q35;hlh-1(cc561) young adults treated with smg-2 or empty vector control RNAi from L1. (G) The number of body movements per minute scored in wild type or hlh-1(cc561) young adults shifted to 25°C at L1. (TIF) [file pgen.1006531.s005.tif]

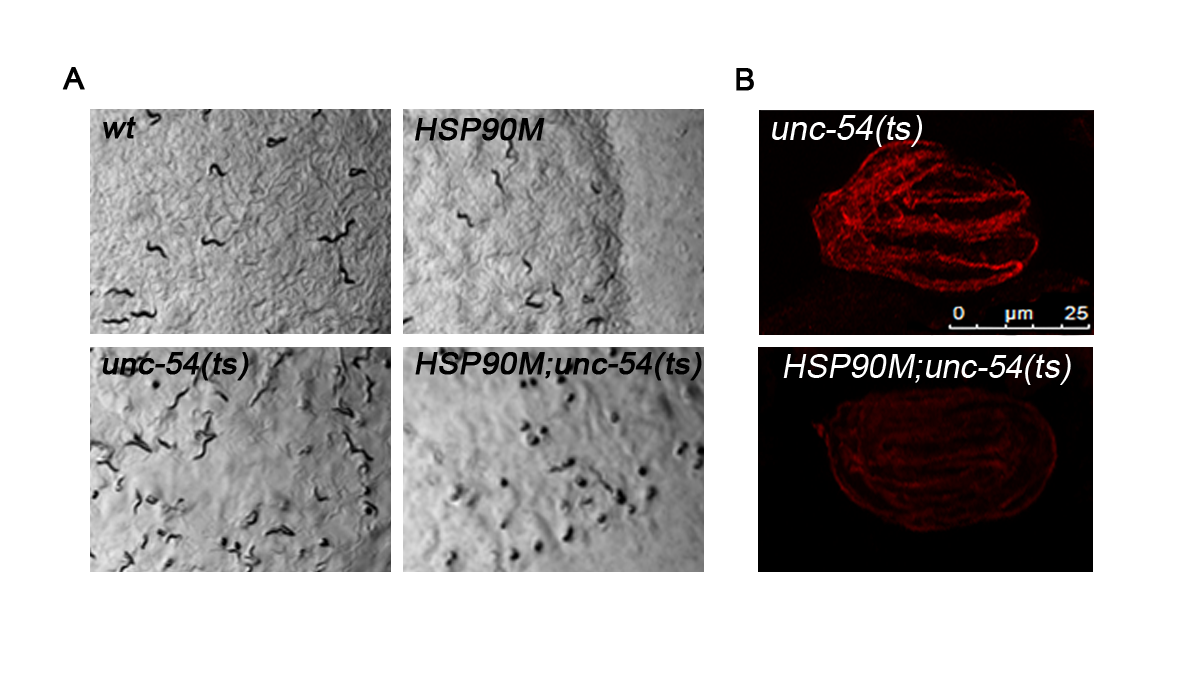

Supplement: S6 Fig — (A) Images of a population of wild type, unc-54(ts), HSP90M or HSP90M;unc-54(ts) embryos laid at 20°C. (B) Representative confocal images (>90%) of unc-54(ts), and HSP90M;unc-54(ts) embryos laid at 25°C and stained with anti-UNC-54 antibodies. The scale bar is 25 μm. (TIF) [file pgen.1006531.s006.tif]

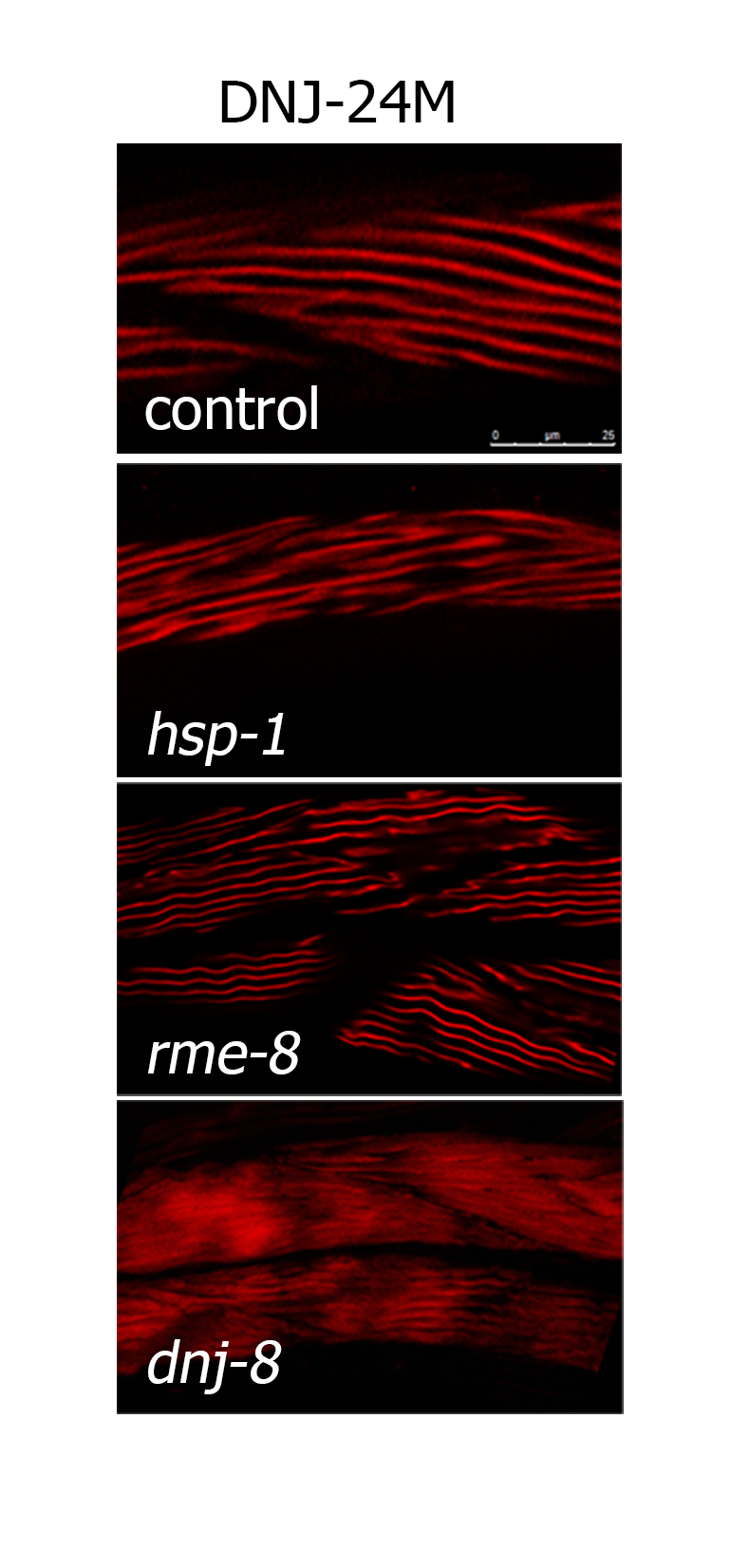

Supplement: S7 Fig — Representative confocal images of age-synchronized DNJ-24M animals treated with control, hsp-1(Hsc70), rme-8(Hsp40) or dnj-8(Hsp40) RNAi and stained with anti-MYO-3 antibodies. Scale bar is 25 μm. (TIF) [file pgen.1006531.s007.tif]
