# Supplementary material for: A Differentiation Transcription Factor Establishes Muscle-Specific Proteostasis in Caenorhabditis elegans
Source: PLoS Genet. 2016 Dec 30;12(12):e1006531. doi: 10.1371/journal.pgen.1006531 (PMC5201269; doi:10.1371/journal.pgen.1006531)
Supplement: S2 Table — (PDF) [file pgen.1006531.s009.pdf]

Table S2. List of strains used in this study.

Strain	Genotype
N2	
KM267	*hsp-16.41::hlh-1*
PD4605	*hlh-1(cc561)*
OH3556	*che-1(ot124)*; *otIs114*[*lim-6p*::GFP + *rol-6(su1006)*]
BC10433	*dpy-5(e907)*; *sEx10433*[*rCesT21B10.7*::GFP + *pCeh361*]
BC12510	*dpy-5(e907)*; *sEx12510*[*rCesT10B5.5*::GFP + *pCeh361*]
CL2070	dvIs70[phsp-16.2::gfp; rol-6(su1006)]
ABZ5	*unc*-54p::YFP; *hsp-16.41::hlh-1*
ABZ1042	dpy-5(e907) I; sEx10433; *hsp*-16.41::*hlh*-1
ABZ1044	dpy-5(e907) I; sEx10433; hlh-1(cc561) II
ABZ1252	dpy-5(e907) I; sEx12510; *hsp*-16.41::*hlh*-1
ABZ1254	dpy-5(e907) I; sEx12510; *hlh-1(cc561) II*
ABZ2072	dvIs70[phsp-16.2::gfp; rol-6(su1006)]; *hsp*-16.41::*hlh*-1
COP705	*knuSiEx94*[pNU315(daf-*21p(mut)*::eGFP), *unc-119(+)*]; *unc-119(ed3); hsp*-16.41::*hlh*-1
COP709	*knuSiEx95*[pNU314(*daf-21p(wt)*::eGFP), *unc-119(+)*]; *unc-119(ed3); hsp*-16.41::*hlh*-1
COP700	*knuSiEx95*[pNU314(*daf-21p(wt)*::eGFP), *unc-119(+)*]; *unc-119(ed3); hsp*-16.41::*hlh*-1
COP708	*knuSiEx95*[pNU314(*daf-21p(wt)*::eGFP), *unc-119(+)*]; *unc-119(ed3); hsp*-16.41::*hlh*-1
COP711	*knuSiEx95*[pNU314(*daf-21p(wt)*::eGFP), *unc-119(+)*]; *unc-119(ed3); hsp*-16.41::*hlh*-1
ABZ152	*knuSiEx94*[pNU375(*hsp-12.2p(mut)*::eGFP), *unc-119(+)*]; *unc-119(ed3); hsp-* [illegible]
ABZ153	knuSiEx94[pNU375(hsp-12.2p(mut)::eGFP), unc-119(+)]; unc-119(ed3); *hsp-*
ABZ154	knuSiEx94[pNU375(hsp-12.2p(mut)::eGFP), unc-119(+)]; unc-119(ed3); *hsp-*
ABZ147	knuSiEx94[pNU374(hsp-12.2p(wt)::eGFP), unc-119(+)]; unc-119(ed3); *hsp*-16.41::*hlh-*
ABZ148	knuSiEx94[pNU374(hsp-12.2p(wt)::eGFP), unc-119(+)]; unc-119(ed3); *hsp*-16.41::*hlh-*
ABZ149	knuSiEx94[pNU374(hsp-12.2p(wt)::eGFP), unc-119(+)]; unc-119(ed3); *hsp*-16.41::*hlh-*
ABZ150	knuSiEx94[pNU374(hsp-12.2p(wt)::eGFP), unc-119(+)]; unc-119(ed3); *hsp*-16.41::*hlh-*
ABZ151	nuSiEx94[pNU374(hsp-12.2p(wt)::eGFP), unc-119(+)]; unc-119(ed3); *hsp*-16.41::*hlh*-1
AM140	rmIs132[punc-54::q35::yfp]
ABZ155	rmIs132[punc-54::q35::yfp]; *hlh-1(cc561)*
AM134	rmIs126[Punc-54::q0::yfp]
ABZ156	rmIs126 [punc-54::q0::yfp]; *hlh-1(cc561)*
AM780	rmIs316[unc-54p::HSP90::GFP;pCFJ90(myo-2p::mCherry)]
DM7350	*pha-1(e2123)*; raEx350 [W03A5.7::GFP + *pha-1(+)* + *rol-6*]
CB1301	*unc-54(e1301)*
ABZ163	rmIs316[unc-54p::HSP90::GFP;pCFJ90(myo-2p::mCherry)]; *unc-54(e1301)*
