# Supplementary material for: A Differentiation Transcription Factor Establishes Muscle-Specific Proteostasis in Caenorhabditis elegans
Source: PLoS Genet. 2016 Dec 30;12(12):e1006531. doi: 10.1371/journal.pgen.1006531 (PMC5201269; doi:10.1371/journal.pgen.1006531)
Supplement: S3 Table — (PDF) [file pgen.1006531.s010.pdf]

Table S3. List of Quantitative PCR primers used in this study.

Gene	Sequence
act-4	F 5'-ATCACCGCTCTTGCCCCATC-3'
	R 5'-GGCCGGACTCGTCGTACTCTTG-3'
bag-1	F 5'-AAGAGGCGGAAAGACATCTG-3'
	R 5'-GATTCCGTTTACGAGCGTCT-3'
C01G10.8	F 5'-TCGTGTTTCTGGCAGTGAAG-3'
	R 5'-AACTTGGCGGATTTGATGAG-3'
C17G10.2	F 5'-CACTGAGTCAGAGCGAGCAG-3'
	R 5'-CGAAATCAAACGGTTTTCGT-3'
cct-2	F 5'-GACAATGCTGGACTTGACTC-3'
	R 5'-CGTAACATCAGCAACCTCTC-3'
cct-5	F 5'-GCGCAACTACTGTTCGATGA-3'
	R 5'-ACGAGCGGCAAGAATATGAC-3'
cct-6	F 5'-CATGATGGACTTCGTGCTGT-3'
	F 5'- CGTCCCTTCAGGTTCTCAAC -3'
che-1	F 5'-GGACAAAATGGAAGTGATCA-3'
	R 5'-TTGGTCGTTGTACTGGAAGA-3'
chn-1	F 5'-AACTGAGCCCAAACGAAGTG-3'
	R 5'-ATGGTAGAGCGCTTTGGAGA-3'
daf-21	F 5'-GCGTGAGAAGGAGGTTGAAG-3'
	R 5'-CCAGATTGGCTTGGTCTTGT-3'
dnj-2	F 5'-TGACGCCAAGCTTTATCTCAT-3'
	R 5'-GTTTCAGCCAGCATTCTCGT-3'
dnj-3	F 5'-ACGAGACAGGAAATTAGAGATG-3'
	R 5'-CAGAAGAACCAGTAGCCCA-3'
dnj-13	F 5'-CGGATAAGAATAAGGAAGCTG-3'
	R 5'- TTCCTTTAGTCCTTCCTCTCC -3'
dnj-15	F 5'-CAAGACCCACGATCACACAA-3'
	R 5'-TTGTCCACTTGTGACTGGTTG-3'
dnj-16	F 5'-GATGAAGCTGTGGCGAAGTT-3'
	R 5'-CGGTTCTTGAGACTTCTGGA-3'
dnj-21	F 5'-GGAGCTCGATATGTCCTTCG-3'
	R 5'-TTGATCGAAACCTCCACGAT-3'
dnj-24	F 5'-CGTCAAGTCGAGTGCACAT-3'
	R 5'-TTGCGTTCTTTCCAGGTTC-3'
F44E5.4	F 5'-GCAAAGCTATTGGTATCGAC-3'
	R 5'-CACGTATGATGGAGTTGTCTTA-3'
fkb-6	F 5'-AGATCGCGGTGATCAATTCT-3'
	R 5'-AATCCGAGCGAATTGTGAAT-3'
hsp-1	F 5'-CTCGAGTCATACGCCTTCAACCTT-3'
	R 5'-GGCCAATCCTTCCAAATCCTTCTG-3'

hsp-12.2	F 5'-ATGTCCGCTATCGAGGTGAC-3'
	R 5'-CGACCTCGAACTTTTCCTTG-3'
hsp-16.2	F 5'-ACTCATGTGTCGGTATTTATC-3'
	R 5'-ACGGGCTTTCCTTGTTTT-3'
hsp-17	F 5'-ATCGTCGTTTTCCACCATTC-3'
	R 5'-ATTGTTTGATCGGCCCAGTA-3'
hsp-25	F 5'-GTCGTGTTGAGGAGGAGAT-3'
	R 5'-AGAAGAAGTGTTCGAGTAGGC-3'
hsp-70	F 5'-ACTCATGTGTCGGTATTTATC-3'
	R 5'-ACGGGCTTTCCTTGTTTT-3'
pph-5	F 5'-TTTCTCGGCACCAAATTATTG-3'
	R 5'-GCAATTTCGGATGTGGAAC-3'
sgt-1	F 5'-CAATCTTGAGGGACACGTT-3'
	R 5'-TCAAGACCAAACGAGTGTTC-3'
sip-1	F 5'-CAATCTTGAGGGACACGTT-3'
	R 5'-CCTTTGGAAGAGTGAACTGG-3'
sti-1	F 5'-AGGAGCTCGAGAAGCAGTTG-3'
	R 5'-TGTAGTGCCTCATCGCTGTC-3'
T07A9.15	F 5'-CTTCTGGACTTTTCTTGCTTG-3'
	R 5'-CCTCAAATAATCTTCCATTTCC-3'
tbc-10	F 5'-TCGACGAGACCGTACCTCTT-3'
	R 5'-AGACGGAACTTCGTTTGCTG-3'
unc-23	F 5'-TGGAAAGGGTGATGAGAAC-3'
	R 5'-TGGGGAGGTTAGTGGAGAT-3'
unc-45	F 5'-GTTAGCAAACACGAAAGAGC-3'
	R 5'-CTTGCAAAGACCAACTAGAGC-3'
unc-54	F 5'-AGGGTATTCAATGGGTCTTC-3'
	R 5'-TCTTCATCAAGCATGGAGAT-3'
